# Temporal dynamics in meta longitudinal RNA-Seq data

**DOI:** 10.1038/s41598-018-37397-7

**Published:** 2019-01-24

**Authors:** Sunghee Oh, Congjun Li, Ransom L. Baldwin, Seongho Song, Fang Liu, Robert W. Li

**Affiliations:** 1 Department of Computer Science and Statistics, Jeju National University,, Jeju City, Jeju Do, S. 690-756 Korea; 2 United States Department of Agriculture, Agriculture Research Service (USDA-ARS), Animal Genomics and Improvement Laboratory, Beltsville, MD 20705 USA; 3 Department of Mathematical Sciences, University of Cincinnati, Cincinnati, OH 45221-0025 USA; 4 College of Food Science and Engineering, Ocean University of China, Qingdao, 266003 China

## Abstract

Identification of differentially expressed genes has been a high priority task of downstream analyses to further advances in biomedical research. Investigators have been faced with an array of issues in dealing with more complicated experiments and metadata, including batch effects, normalization, temporal dynamics (temporally differential expression), and isoform diversity (isoform-level quantification and differential splicing events). To date, there are currently no standard approaches to precisely and efficiently analyze these moderate or large-scale experimental designs, especially with combined metadata. In this report, we propose comprehensive analytical pipelines to precisely characterize temporal dynamics in differential expression of genes and other genomic features, i.e., the variability of transcripts, isoforms and exons, by controlling batch effects and other nuisance factors that could have significant confounding effects on the main effects of interest in comparative models and may result in misleading interpretations.

## Introduction

Similar to microarrays, investigators have been increasingly conducting experiments that focus on ontological alterations across a series of time periods^1–8^. Perhaps even more popular is the use of longitudinally repeated measurements at different time points in relation to some baseline stimuli or perturbation. Prior to the main downstream analyses, a prerequisite step must be the removal of experimental artifacts and unwanted sample-to-sample variation using appropriately proposed methods in pipelines. While this has long been recognized as an important step in the analysis of high-throughput data, it has largely been overlooked in the detection of significantly differential expression^9–18^.

The purpose of this research is to develop data management procedures for the increasing wealth of data being generated by new approaches and to deepen the characterization of temporal dynamics by including isoform diversity in addition to gene-level analyses. In this study, we describe how to incorporate improved strategies to remove systematic biases and to fully characterize temporal dynamics by accounting for data-driven inherent features. This is based on our large-scale time course longitudinal stimuli-response data at every step, along with a panorama snapshot of the entire workflow (Supplementary Fig. S1).

## Results

### Exploratory and differential expression analysis in temporal dynamics

**Figure 1:**
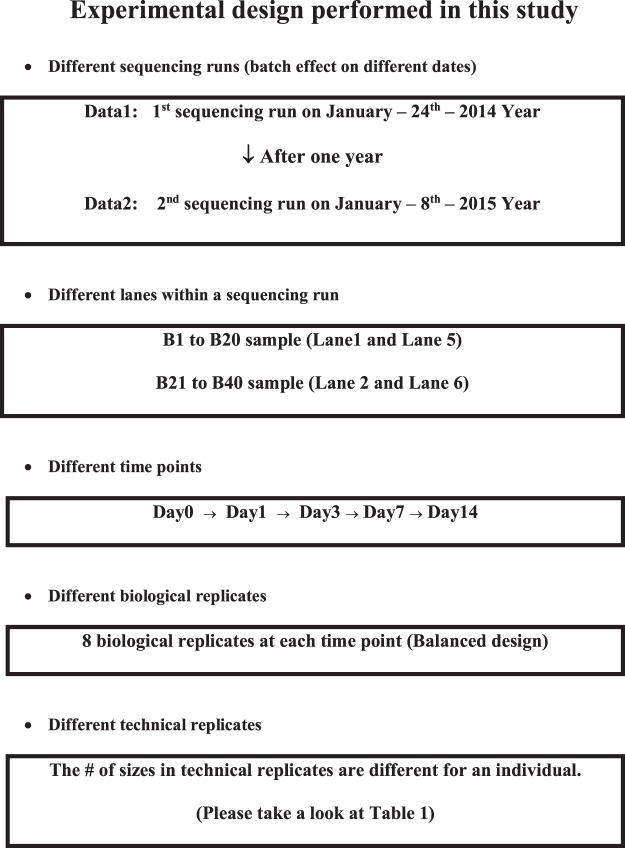
Description of experimental design performed in this meta study.

Our meta-framed longitudinal data have been sequenced twice in different sequencing dates and temporal expression levels have been measured over the 5 distinct time points, from Day 0 (D0) through Day 14 (D14). And 8 biological replicates at each time point have been utilized in this study. To characterize the complexity of temporal dynamics, our proposed Bayesian dynamic AR method with batch correction and isoform diversity, compared to existing static and other dynamic methods have been employed. A schematic illustration of the entire analytical strategy in the detailed analytical pipelines at each window is depicted in the section of 4. METHODS and in Supplementary Fig. S1. A description of the samples in the experimental design is also presented in the section of 4. METHODS, Fig. 1 and Dataset S1.

#### After pooling replicates for a biological individual sample at each time point

**Figure 2:**
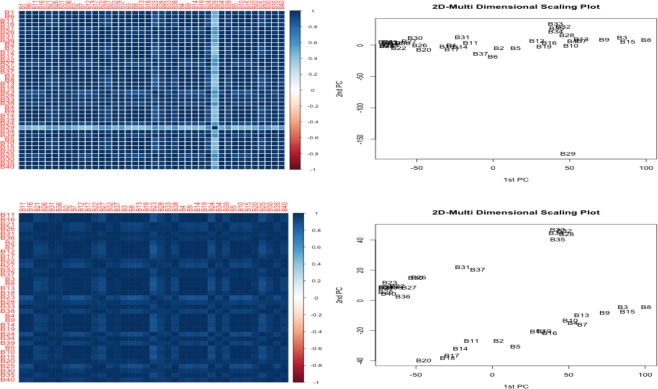
(1) and (2) represents correlation heatmap and multi-dimensional plot with B29 sample after pooling replicates of genes (3) and (4) represents correlation heatmap and multi-dimensional scaling plot without B29 sample after pooling replicates of genes, respectively.

Based on the results where the first pipeline was used to analyze our raw data, without regard to the sophisticated diagnosis in the exploratory analyses (See Supplementary Fig. S1), we observed very low sensitivity for detection of temporally differentially expressed genes in Dataset S3-(1), despite the high quality of the sequencing samples in Dataset S1 and S2, and Fig. 2. These observations can be explained by several possibilities, including the impact of outlier samples, pooling issues without adjustment for batch effects on different sequencing dates or other experimental technical replicates, normalization issues, exploratory analysis-free preprocessing steps, and the selection of inappropriate differential expression methods, resulting in large confounding effects of batch and other experimental nuisance factors in comparative models between baseline time point versus later time points.

**Figure 3:**
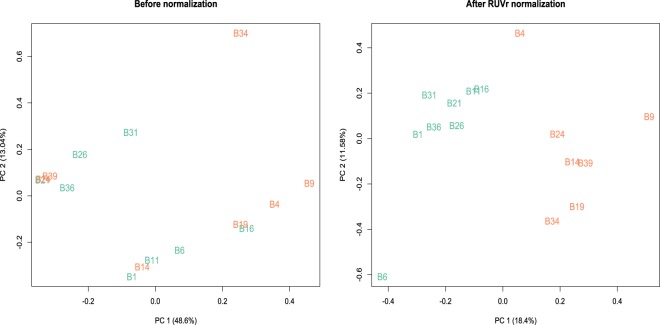
PCA plot after pooling replicates before and after correction of systematic biases in pairwise comparisons of time points (baseline time point D0 versus D7) without sample B29, respectively.

Hence, we excluded sample B29, which was highly suspected to be an outlier, as shown in Fig. 2, and we also employed dynamic specific methods for pooled data in analytical pipeline 2. The objective of this pipeline was to evaluate whether there was any significant effect of sophisticated exploratory analysis with sample diagnostic tools and the use of dynamic specific methods, compared to pipeline 1. Based on the normalization strategies in edgeR and DESeq.^13,19–21^, our data still showed the extra variability of samples, indicating that there could be other significant lurking factors in the data that would need further correction of systematic artifacts (Left panel of Fig. 3). In Fig. 2, after the removal of sample B29, we saw a distinguished pattern that was more likely grouped with samples within each lane than by time point, implying a substantial lane effect between samples (B1 to B20 versus B21 to B20) as is shown in the 2D multidimensional scaling plot. Furthermore, we confirmed the unwanted systematic artifacts due to different technical lanes by using a 3D movie of a principal component analysis plot (Supplementary Movie S1).

In this study, our experimental dataset is a meta-framed longitudinal time course with repeated measurements, i.e., a within-subject stimuli-response dataset. In principle, within-subject longitudinal stimuli-response data is applied to a Bayesian dynamic autoregressive model (AR), which more precisely targets the repeatedly measured time course RNA-Seq data, as was proposed in our previous study^3,5^. Another dynamic-specific method, maSigPro, which is implemented in between-subject factorial time courses and is designed for the comparison of cases (single or multiple cases) versus control group over time, was also conducted for our within-subject time series data for a comparison. The latter method showed less sensitivity than our proposed Bayesian dynamic autoregressive model because our experimental setting is designed in a within-subject stimuli-response (data not shown).

Conclusively, when performing differential expression analysis with well-qualified samples after correction for systematic artifacts and making use of the desired dynamic method that was implemented for longitudinal data, we clearly detected more highly significant genes and isoforms that are insignificant in pipeline 1 due to the significant lurking factor (lane effect) in our experiment and confirmed in the multiple results (Dataset S3 and Supplementary Fig. S2). And more specifically, for Bayesian dynamic AR method, the detected genes (before correction of unwanted biases) are all included in those genes (after correction) as the nested sets (Supplementary Fig. S2-(3) to S2-(10)). In addition, the correction of systematic artifacts and filtering out sample B29 revealed temporal patterns in 147 and 251 genes (detected by edgeR and DESeq, respectively) between the stimulated D7 and control D0 groups in Supplementary Figs S2-(1) and S3, indicating a higher sensitivity in differential expression analysis with the improved analytical pipeline.

**Figure 4:**
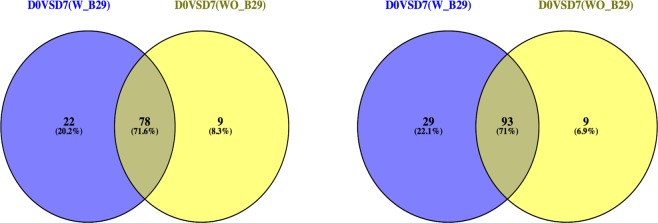
Comparison before correction of unwanted biases with and without B29 when comparing D0 versus D7 at the tail probability of 0.1 after pooling samples for the Bayesian dynamic AR model of genes and transcripts, respectively.

Interestingly, when compared to naïve static methods, the advantageous nature of our proposed Bayesian dynamic AR method has the capability to detect significantly differentially expressed genes before correction. This suggests that our Bayesian dynamic AR model explicitly captures the variability of replicates within a group and the extra variability due to the experimental systematic artifact of lane effect on the given data. In other words, as shown in Dataset S3-(2), S4-(2) and Fig. 4, most of the genes detected by static methods after correction are overlapped with the list of our dynamic method before correction.

Moreover, another advantageous property of our Bayesian dynamic AR model compared to other employed static methods in this study is that the majority of differentially expressed genes and isoforms are more often detected in highly expressed patterns than in zero and low-expressed noise sets. Generally, the Fisher exact pairwise static test, which is a robust simple pairwise static method, also tends to identify more likely highly expressed genes, as is confirmed in previous studies^22,23^. Therefore, we confirmed in this study that the temporal dynamic genes significantly identified by the Bayesian dynamic AR model have mostly been included in the list from the static Fisher exact test, which was based on before-correction data (Dataset S3-(1) and Dataset S4).

#### Before pooling replicates for an individual biological sample at each time point

At the beginning of this study, we expected to find a significant batch effect between different sequencing dates (See the labels of samples in the batch column in Dataset S1). However, the variability between the different lanes was much greater than that of the batch effects of two different sequencing dates. For example, the correlation values among samples within each lane at the baseline time point, Day0, represent the overall very high reproducibility of greater than ~0.9, the values of the reproducibility of samples B1_L1_1 versus B21_L2_1, representing two different lanes at Day0, dramatically drops to less than ~0.5 (See Dataset S2 and Supplementary Fig. S4). This confirms the extra variability of samples due to the systematic artifact of lane effect between samples B1 to B20 versus B21 to B40. Compared to this explicit extra variability of samples in correlation metrics on unpooled data based on pipeline 3 (Supplementary Fig. S4), the lane effect was implicitly a lurking pattern in the pooled data (Fig. 2 and Dataset S2-(2)). That is, see the correlation heatmaps (after pooling replicates) in Fig. 2 and corresponding heatmaps (before pooling) in Supplementary Fig. S4 simultaneously.

In summary, our integrated meta longitudinal dataset shows that technical variability in library prep exerts a much greater effect compared to batch effects between different sequencing runs with a time-lag. This suggests that such unwanted nuisance factors should be removed prior to subsequent major analyses to reduce significant confounding effects on the main effects of interest and misleading results of statistical testing of the model^14–18,24^. In the analyses of pipeline 3, we first examined the effect of the correction of systematic artifacts using the static methods, edgeR and DESeq. These methods showed less sensitivity for truly differential expression before the correction compared to certain sets of differential expression identified after correction. In particular, edgeR tends to more often identify falsely differentially expressed genes that have zero expression for most samples than does DESeq when analyzing data that contains unadjusted biases and unpooled replicates, as in Dataset S3-(3) and Supplementary Fig. S5. Regarding the impact of the outlier sample B29, overall the D0 versus D7 comparison with sample B29 identifies nearly three times more differentially expressed genes and isoforms than without this sample, indicating the higher false calls in differential expression analysis (Dataset S3-(3) and S3-(4)).

Importantly, for unpooled data, a biological sample is split into subdivided technical replicates with varied sizes between two consecutive time points (baseline versus stimulated group) (See the detailed different number of technical replicates in unpooled data from Dataset S1). Hence, it is not meaningful to systematically apply our proposed Bayesian dynamic AR model in longitudinal settings on unpooled data. However, pipeline 3 confirmed that the more significant effect of systematic unwanted biases due to lane effect than pooled data has been observed in Fig. 2 versus Supplementary Fig. S4. Additionally, there are currently no computational methods that incorporate those effects in lower-level preprocessing procedures prior to downstream analyses. Taken together, we simply pool technical replicates of multiple *.sam (or *.bam) files to increase the power of mapped read counts for an individual biological sample after correction of systematic artifacts for our proposed Bayesian dynamic AR method.

#### Quantitative Real-time Reverse Transcription (RT) PCR

**Figure 5:**
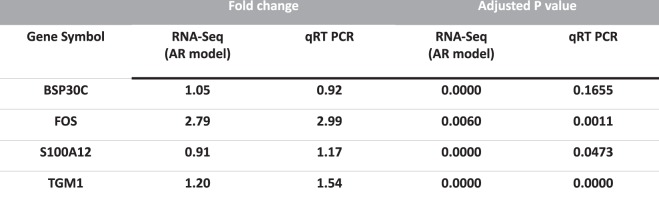
qRT PCR validation for selected gold standard gene list that has been detected by Bayesian dynamic AR method, but not by other static methods.

**Figure 6:**
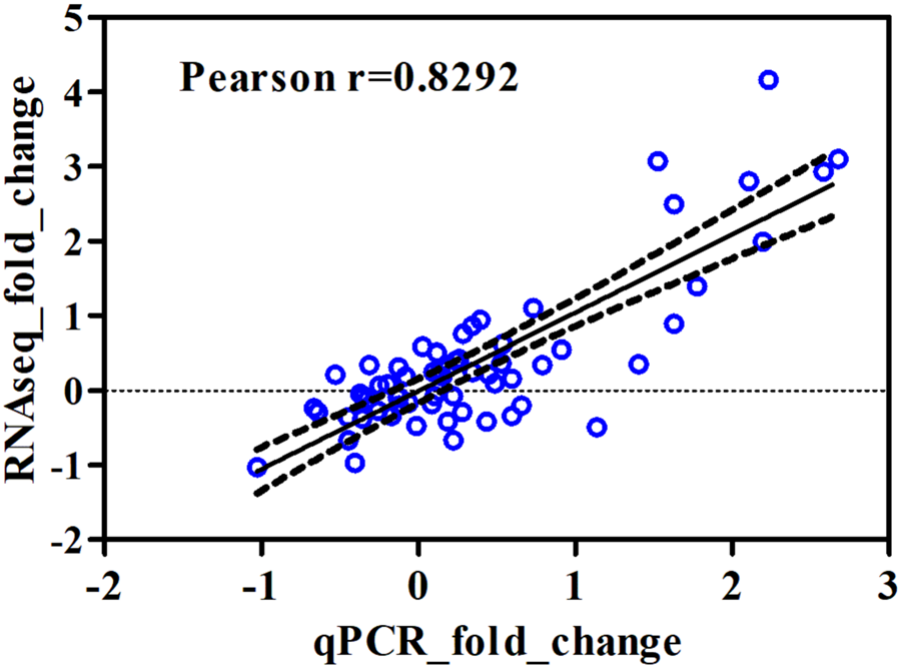
Pearson correlation value between the log2-transformed fold changes of RNA-Seq data and qRT-PCR validation. Each dot (in blue) represents a data point for the selected genes and each corresponding sample (4 genes and 8 replicates in control (D0) and later time point, D1 and D14, respectively). And the dashed lines are 95% confidence interval.

Our proposed Bayesian dynamic AR specific differential expression that has not been detected by other static and dynamic methods has been validated with qRT-PCR validation procedure whether those differential expression patterns are truly temporally differentially expressed genes in terms of biological perspectives or not. Two selected genes for each comparison (D0 versus and D1 and D14, respectively) that have been uniquely identified using the AR model were validated independently by qRT-PCR (Fig. 5 and Dataset S7). Our selected genes confirmed the highly significant correlation between AR specific differential expression and validation results, suggesting that the higher sensitivity when using AR method in differential expression analysis and after correction of unwanted systematic artifacts that could be significant confounding effects in differential testing. For example, comparing to time zero (D0), a 14-day post-infusion (D14) induced a significant change in the mRNA expression of FOS (*P* < 0.01), in a good agreement with the AR-based RNAseq results. There existed a strong positive correlation between log2 transformed fold changes calculated from the normalized hit counts (RNAseq) and those derived from the gene copy numbers detected by qRT-PCR (Correlation coefficient R = 0.8292, *P* = 4.71 × 10^−17^) as shown in Fig. 6. Moreover, we also further validated the gene list that has been identified by our proposed dynamic AR method and after correction of unwanted systematic biases with our previous study using simple pairwise static method^25^ (Dataset S7). The highlighted genes represent the overlap between current and previous study.

### Differential exon and isoform diversity in temporal dynamics

To identify various temporal patterns at the isoform level, we applied the most popular and robust methods, Cuffdiff and DEXSeq.^3,26–43^. Datasets S3-(1), S3-(2), S6-(1) and S6-(2) represent the analysis of differential expression between a reference time point versus later time points for pooled and unpooled data at the transcript isoform level to investigate the impact of correction (before- and after-correction), respectively.

Analogous to gene level analyses before the correction of the systematic artifact of lane effect, for both exon and isoform level analysis, relatively few significant sets were identified by the existing static Cuffdiff and DEXSeq methods, implicating suspected confounding effects (Dataset S3-(1) and Supplementary Movie S2). Therefore, we also performed differential expression at the exon and transcript levels by the static edgeR and DESeq methods and our dynamic AR model, which enables incorporation of other nuisance factors. Thus, after correction, we detected more significant sets of exons and transcripts (Datasets S3-(1), S3-(2), S4-(1) and S4-(3)). The analyses of transcripts and individual exons for each gene demonstrated that, as expected in the complex architecture of gene-to-multiple isoforms, significant genes do not necessarily guarantee significance for all isoforms of those genes, and vice versa. Additionally, the magnitude of the expression level of a single gene is not similar for all of the corresponding multiple isoforms, implicating the variety of the expression spectrum for each isoform, with exon- and isoform-specific alterations. Thus, the complete features of the temporal dynamics of transcriptomes in gene regulation should be explored within gene-isoform families, because a parent (i.e., gene) is linked with multiple children (i.e., isoforms) to generate distinct functionalities and protein structures.

### Gene set enrichment analysis and network modules

To explore the biological processes affected and functional mechanisms involved, we further conducted gene set enrichment analysis and network analysis on the temporal dynamic gene lists obtained from differential expression analyses^7,44–50^. We carried out these functional analyses based on the data after correction of unwanted biases in both static and dynamic methods by pipeline 2, using pooled data (Dataset S5 and Supplementary Figs S6 and S7). Based on the dynamic data, the extracellular exosome and matrix, chromatin remodeling, DNA-dependent transcription, initiation, hemopoiesis, and RNA binding were significant for D0 versus D1. For the differentially expressed genes in D0 versus D14, the DNA-dependent transcription and initiation, detection of stimulus, nervous system development, and immune response terms were highly significant. Homopoiesis, chromatin remodeling, and Parkinson’s disease were consistently detected for D0 versus D1 through D14.

## Discussion

We have provided generalized analytical pipelines for meta-framed longitudinal RNA-Seq data in the above sections. It is of primary importance to correct for unwanted systematic artifacts due to the various types of experimental lurking factors, such as lane effects and other batch effects, in combined samples to increase the power of detection. Additionally, it is well known that aberrant patterns of splicing events are highly associated with tissue-, external condition-, and developmental stage-specific alterations^3,26,28–30,33–35,37–40,42,43,51–56^. Thus, state-of-the-art pipelines facilitate a more complete landscape to better characterize the complexity of temporal dynamics by removing unwanted systematic biases, such as experimental artifacts, by incorporating multiple factors contained in experiments and by defining isoform diversity in our proposed Byaesian dynamic AR model for temporal dynamics.

Despite this importance to correct systematic artifacts in combined meta-framed data prior to major downstream analyses, such as differential expression analysis, in general, each method has unique input and output data, distinct normalization methods and rationale for differential expression analysis. It is timely that a universal analytical pipeline outlining a concrete plan for analytical steps should be proposed, as increasingly complicated datasets and combined metadata have become available in the postgenomic era. Indeed, this pattern will continue to grow with the continued rapid development of new technologies, and as an ever-greater amount of data is being generated, which is leading to a higher-order of data entropy.

Thus, it is certain that the issues have received little attention in more complicated experiments such as longitudinal within-subject and factorial between-subject stimuli-response data^57–59^. Hence, our deeper level analyses of temporal dynamics of meta-framed RNA-Seq data with longitudinally measured stimuli-response measurements will accelerate the way in which to more precisely identify time-specific and condition-specific multiple external factors of stimuli, as well as tissue- or disease-specific differentially expressed genes and other genomic features. Our methods also provide new insights into analytical pipelines by defining biomarker candidates that are highly relevant to aberrant patterns of functionalities, network modules, and pathways in global temporal dynamics in a variety of clinical and experimental settings, including integrated meta time course data and disease spectrum progressive models.

There still many issues to be further addressed in a variety of more complex experimental designs, such as temporal RNA-Seq data, and especially in combined time course RNA-Seq datasets, in terms of the development of computational methods in the field of community. Examples include significant artifacts such as batch issues^14–18^, sophisticated normalization^12,60,61^, and several different types of experimental designs for time points^2,5–8,14,16,57–59,62^ (e.g., short, moderate, or long time series, or those containing multiple external factors at each time point, or not). Additional issues include whether the data is a single or multiseries factorial time course dataset, a circadian rhythmic periodical dataset, a single cell cycle dataset, or a meta-framed time course. Important is the establishment of a gold-standard benchmarking list in databases^63^.

As an extension of this study, we are currently developing Bayesian dynamic models to define temporal dynamics for between-subject stimuli-response in factorized RNA-Seq time course experiments to better take into account systematic biases, multiple experimental factors, and isoform diversity in a model of differential expression in R and OpenBUGS (Winbugs) (implementation is underway). Another promising study in temporal dynamics would be to characterize a progressive disease model, such as pediatric cancer progression, by targeting initiation, progression time points and period, perturbation of cancer progression, and estimation and prediction of unobserved time points^64–75^.

More specifically, complete analytical pipelines for noncoding RNAs (ncRNAs) are also needed, as aberrant patterns in (very) long and noncoding RNAs have been explored as important biomarkers for the classification of subtypes of cancers and other diseases, as primary factors in oncogenesis, and for therapeutic effects^69,71,72,76^.

## Methods

### Autoregressive model (AR) incorporated with systematic artifacts and isoform levels

To precisely characterize temporal dynamics in within-subject stimuli-response longitudinal RNA-seq data in the format of a single series time course experiment, we employed an autoregressive model (AR) that had been initially proposed to account for the count property with Poisson gamma (negative binomial distribution) and time dependency in our previous study^77^. To identify differentially expressed genes between the baseline time point (D0) with ruminal infusion of butyrate versus later time points in the comparative tests, that is, D0 vs D1 (to D14), we computed the tail probability of (*φ*_*i*_|${y}_{i}$) of gene i, such that p (*φ*_*i*_ > 0|${y}_{i}$) or p (*φ*_*i*_ < 0|${y}_{i}$) for i = 1,…, n using Monte Carlo Markov Chain (MCMC) with 10,000 simulations and 8,000 burn-ins, where *φ*_*i*_ denotes the autocorrelation in the Poisson gamma AR model, and ${y}_{i}$ represents the expression levels for i-th gene (see the further detailed explanation of our proposed model)^77^. The cutoff value of 0.1 to tail probabilities in this model is selected properly and corresponds to a 0.1 false discovery rate (FDR) for other methods. It indicates the significance of differential expression for each gene-by-gene testing. This method is able to be straightforwardly extended to detect temporally differential expression for the quantification level of isoforms and other genomic features. Strikingly, our proposed dynamic model enables the inclusion of the factor of systematic biases from lane effects, which was estimated from preprocessing procedures in the differential expression analysis. Furthermore, this dynamic model has the capability to infer multiple factors in various experimental and clinical settings simultaneously, such as other additional nuisance factors resulting in unwanted systematic biases in a given dataset.

### Comparison with other methods

Prior to down-stream analyses, the following diagnostic analyses on all samples for both the before and after pooling data were performed in the R package with the latest versions: gplots, RUVSeq.^78^, multivariate analyses, and Venny online tool. For comparison to our proposed temporal dynamics Bayesian AR method, differential expression analyses of genes, including pairwise comparisons, a generalized linear model incorporating multiple factors, and dynamic methods were carried out using Cuffdiff, the Tuxedo Suite, edgeR, DESeq, voom in limma, and maSigPro, in turn^1,19,77,79–85^. For the quantification of isoforms (and exons) and the identification of differential expressions of splicing, Cuffdiff and DEXSeq were employed^8,21,85,86^. For functional analyses, we employed Networkanalyst and David^87,88^.

### Experimental design and preprocessing analysis

#### Experimental design

Four Holstein cows were ruminally cannulated, as previously described^31,89–94^. Cows in mid-lactation were fed ad libitum a total mixed ration consisting of 50% corn silage and 50% concentrate on a dry matter basis. The cows were moved to a tie stall barn for adaptation and acclimation 7 d prior to the experiment. A ruminal infusion of butyrate was initiated immediately following the 0 h sampling (baseline control) and thereafter continued for 168 h at a rate of 5.0 L/d of a 2.5 M solution (representing >10% of the daily anticipated metabolizable energy intake to support lactation) in a buffered saliva solution (pH 7.0; 3.8% KHCO3, 7.3% NaHCO3) as a continuous infusion. After 168 h of infusion, the infusion was stopped and the cows were maintained on the basal lactation ration for an additional 168 h for sampling. Rumen epithelial samples were serially collected via biopsy through the rumen fistulae at 0, 24, 72, and 168 h of infusion, and at 24 and 168 h post infusion (post 24 h and post 168 h, respectively). The ruminal pH was monitored using a standard pH meter and recorded at each sampling. Rumen epithelial samples were snap frozen in liquid nitrogen and stored at −80 °C until RNA extraction (See Dataset S1). All animal care and handling was conducted according to the guidelines approved by the USDA Beltsville Area Institutional Animal Care Committee.

#### RNA extraction and sequencing using RNA-Seq

Total RNA was extracted from 24 rumen epithelial samples using Trizol (Invitrogen, Carlsbad, CA, USA), followed by DNase digestion and Qiagen RNeasy column purification (Qiagen, Valencia, CA, USA). The RNA integrity was verified using an Agilent Bioanalyzer 2100 (Agilent, Palo Alto, CA, USA). High-quality RNA (RNA integrity number (RIN) > 8.0) was processed using an Illumina TruSeq RNA sample prep kit following the manufacturer’s instructions (Illumina, San Diego, CA, USA). After quality control procedures, individual RNA-seq libraries were pooled based on their respective sample-specific 6-bp adaptors and sequenced at 50 bp/sequence read using an Illumina HiSeq. 2000 sequencer, as previously described^25^.

#### Preprocessing and exploratory analysis

All raw sequence fastq files were initially preprocessed against the reference genome, Bos_taurus.UMD3.1.80.gtf, downloaded from tools^95,96^. In the sample raw data, biological replicates were collected from 8 different cell lines and technical replicates were run in different sequencing dates (Oct-24-2014 and Jan-08-2015) and in distinct lanes. The tuxedo method, bowtie, TopHat, and Cufflinks tools for trimming fastqc were performed using the latest version of tools. For the quantification of expression levels, there were two different types of the pipeline that were compared in this study: after and before pooling of the replicates. Hereafter, we refer to these as after and before pooling data. In the pipelines, the mapped expression FPKM levels quantified by Cufflinks were further utilized for the purpose of detecting temporally differentially expressed genes and isoforms. Prior to the identification of temporally expressed genes and isoforms, we employed exploratory analysis for all individual samples to verify sample reproducibility, variability, and unwanted systematic biases by making use of the diagnostic tools^12^.

#### Quantitative Real-time Reverse Transcription (RT) PCR

Purified total RNA samples were converted to cDNA using an iScript Advanced cDNA Synthesis Kit (Bio-Rad, Hercules, CA, USA). *Quantitative Real-time Reverse Transcription (RT) PCR* analysis was performed using an Absolute Quantitation (standard curve) method. Briefly, the reaction was carried out in a SsoAdvanced Universal SYBR Green Supermix (Bio-Rad) using 200 nM of each amplification primer and 100 ng (the input total RNA equivalents) of the first-strand cDNA in a 25 μl reaction volume as previously described^97,98^. Real-time amplification was conducted on a CFX96 Real-Time PCR Detection System (Bio-Rad) with the following profile: 95 °C for 120 s, 40 cycles of 95 °C for 30 s, 60 °C for 30 s and 72 °C for 30 s followed by a melting curve analysis for each primer pair. Standards with known quantities (copy numbers) for a single mRNA sequence (gene of interest) were prepared from PCR products purified using Agencourt AMPure XP beads (Beckman Coulter, Indianapolis, Indiana, USA). The expression levels were determined from a standard curve of known target cDNA copy numbers (1.0 × 10^1^ to 1.0 × 10^5^ molecules per reaction) and analyzed simultaneously with unknown experimental samples on the same plate. The primers used in the study were listed in Supplementary Table S6.

### Development of the analytical pipeline

Pipeline 1: Initially, we pooled all of the technical replicates from different lanes and sequencing dates in the preprocessing procedures, resulting in eight biological samples at each time point, identical to the previous study^99^. For the detection of temporally differentially expressed genes and isoforms, we intuitively performed typical pairwise comparison methods, as our main hypothetical testing of interest is to identify any significant changes between treatment groups (later time points, D3 through D14) versus the baseline control group (Day 0). The comparative methods were carried out by Cufflinks and Cuffdiff, edgeR, and DESeq pairwise comparison. We utilized FPKM (fragments per kilobase per million mapped reads) values as the input data of expression values for the direct comparison of methods. When pooling replicates, we clearly observed that the quality of sample B29 was not good due to the sample and library prep. Further examination of this outlier sample explored how discrepant results impact downstream analyses, such as differential expression analysis, when this sample was either excluded or included. This step in our study ensured that poor quality samples were carefully dealt with in the preprocessing procedure as a prerequisite step since these samples can significantly affect the following results of downstream analyses.

Pipeline 2: As described in pipeline 1, our experimental design attempted to characterize the stimulated alterations across different time points from D0 through D14, and each individual sample was longitudinally observed as the repeated measurement during the given time period. Since the comparison in our study was focused on the D0 baseline time point versus each of the later time points, as the format of simple pairwise comparisons (i.e., before and after stimulus), other than a full time course series, simple pairwise static methods under the independent assumption of samples before and after stimulus might also work to some degree. However, it was evident that the expression levels were highly correlated between two neighboring time points, as the samples were longitudinally measured after an external stimulus of ruminal infusion. Additionally, our analysis also shows time-dependent expressed patterns between consecutive time points, as shown in Supplementary Fig. S2-(2), such that previously differentially expressed genes tended to also be significantly identified at the current time point. Hence, in order to better characterize data-driven features, for this pooled dataset we also performed dynamic-specific methods (as well as static pairwise comparisons) that are more precisely implemented for our within-subject stimulus-response longitudinal data, containing time-dependent structures^1–5^. In addition to sample B29, we also observed extra variability among samples due to the distinct lanes, indicating a significant lane effect in the experiment. Prior to the main downstream analyses, we adjusted the systematic artifacts that could affect the main biological factors of interest (i.e., the time factor), as shown in the study that explored unwanted biases in static data^12^, and we further incorporated the effect in our proposed Bayesian dynamic AR method. In other words, to examine how systematic biases can be confounding in the detection of differentially expressed genes and isoforms in this pipeline, we compared the results of differential expression with and without correction of the extra variability of samples before executing the main differential expression analysis. Furthermore, we also performed gene ontology, gene set enrichment analysis, and network module analysis based on the temporal dynamics of gene and isoforms as putative biomarkers.

Pipeline 3: This pipeline aims at exploring whether there are unwanted systematic biases that significantly affect the main factors of biological interest in unpooled samples. Unlike the previous pipelines that directly analyze the pooled data, prior to pooling all of the replicates we carefully examined all samples with diagnostic tools in an exploratory analysis, as described in the statistical analyses section^12^ (See also Supplementary Fig. S1 and Dataset S1). In this pipeline, we explore how the nonnegligible effect of outlier samples and extra sample to sample variation due to lane effects behaves in data that contains unpooled replicates. If any unwanted bias is significantly present in the exploration of samples, this effect should be corrected prior to pooling the samples. We then perform typical pairwise static methods by incorporating those effects into the models of differential expression. The purpose of this is to test whether the effect of correction on systematic biases in the preprocessing steps affects the detection outcomes of temporally differentially expression of unpooled data. We examined the results that were obtained both from before and after the correction of systematic biases and how (in)consistently they present in differential expression analysis.

## Supplementary information

Supplemental Information

Dataset S1

Dataset S2

Dataset S3-(1)

Dataset S3-(2)

Dataset S3-(3)

Dataset S3-(4)

Dataset S4-(1)

Dataset S4-(2)

Dataset S4-(3)

Dataset S4-(4)

Dataset S5

Dataset S6-(1)

Dataset S6-(2)

Dataset S7

Supplemental Vide S1-(1)

Supplemental Vide S1-(2)

Supplemental Video S2-(1)

Supplemental Video S2-(2)
