# Supplementary material for: Temporal dynamics in meta longitudinal RNA-Seq data
Source: Sci Rep. 2019 Jan 24;9:763. doi: 10.1038/s41598-018-37397-7 (PMC6345883; doi:10.1038/s41598-018-37397-7)
Supplement: Supplementary file 1 — Supplemental Information [file 41598_2018_37397_MOESM1_ESM.pdf]

Supplementary Information

Temporal dynamics in meta longitudinal RNA-Seq data

Sunghee Oh*[1], Congjun Li[2], Ransom L. Baldwin[2], Seongho Song[3], Fang Liu[2,4] and Robert W. Li*[2]

Affiliations:
[1]Department of Computer Science and Statistics, Jeju National University, Jeju City, Jeju Do, S. Korea, 690-756
[2]United States Department of Agriculture, Agriculture Research Service (USDA-ARS), Animal Genomics and Improvement Laboratory, Beltsville, MD 20705, USA
[3]Department of Mathematical Sciences, University of Cincinnati, Cincinnati, OH, 45221-0025, USA
[4]College of Food Science and Engineering, Ocean University of China, Qingdao, China, 266003

***Correspondence should be addressed to Dr. Sunghee Oh, sshshoh1105@gmail.com and Dr. Robert W. Li, robert.li@ars.usda.gov**

Running title: Characterization of integrated longitudinal RNA-Seq data

Keywords: RNA-Seq, stimuli-response longitudinal data, temporal dynamics, metadata, systematic artifacts, isoform diversity

Bayesian Dynamic AR Method Procedure

< # I. Manual with an example data For Mac OS X Users >

```
# Sunghee$ R CMD INSTALL /Users/Sunghee/R2OpenBUGS_3.2-3.2.tar
library(R2OpenBUGS)
# library(R2WinBUGS)

############################################################################
# To set the WINE working directory and the directory to OpenBUGS
# To change the OpenBUGS.exe location as necessary
############################################################################

WINE="/opt/local/bin/wine"
WINEPATH="/opt/local/bin/winepath"
OpenBUGS.pgm="/Users/sungheeoh/.wine/drive_c/Program
Files/OpenBUGS/OpenBUGS323/OpenBUGS.exe"

############################################################################
# I-(1) Simple auto-regressive poisson model with MCMC algorithm in OpenBUGS
# Prior to incorporating replicate factor and systematic artifact of lane effect
############################################################################

sink("AR-1.txt")
cat("
model{
	for (i in 1:I) {
	   for (t in 1:T) {
	      Y[i, t] ~ dpois(mu.y[i, t])
	      mu.y[i, t] <- exp(beta.i[i] + w.it[i, t])
	   }
	}
	for (i in 1:I) {
	   beta.i[i] ~ dnorm(0, 1000)
	   pi.1[i] ~ dunif(-1, 1)
	}
	sig2 <- 1/tau2
	tau2 ~ dgamma(0.05, 0.05)
	for (i in 1:I) {
	   sig.w1[i] <- sig2/(1 - pi.1[i] * pi.1[i])
	   w.it[i, 1] ~ dnorm(0, sig.w1[i])
	   for (t in 2:T) {
	      mean.wit[i, t] <- pi.1[i] * w.it[i, t - 1]
```

```
            w.it[i, t] ~ dnorm(mean.wit[i, t], sig2)
          }
        }

      }",fill=TRUE)
sink()

#################################################################################
# To read data
#################################################################################

directory1 <- "/Users/sungheeoh/"
data.expr <- read.csv(paste(directory1,"D0VSD1_FOR AR.csv",sep=""),header=T,as.is=T)
anno <- data.expr[,1:6] #annotation
expr <- data.expr[,-c(1:6)] #expression data
dim(expr)
head(expr)

#################################################################################
# In order to more efficiently run our proposed AR model
# As it is gene by gene testing,
# We recommend to filter out genes with zero or low expression in advance
# Resulting in <  less than ~ 10,000 genes (or isoforms)
# If the size of RAM is large enough,
# It allows to run the full list of genes (or isoforms)
#################################################################################

library(edgeR)
table(rowSums(expr==0)==ncol(expr))
cpm <- cpm(expr)
lcpm  <- cpm(expr, log=TRUE)
filtered.data <- rowSums(cpm>1)>=ncol(expr)
filtered.expr <- expr[filtered.data,]
filtered.anno <- anno[filtered.data,]
dim(filtered.expr)

# No. of genes (or isoforms)
I <- nrow(filtered.expr)
# No. of time points in comparison of temporal changes
T <- 2
# No. of time points in comparison of replicates
R <- 8
Y <- cbind(apply(filtered.expr[1:I,1:R],1,mean),apply(filtered.expr[1:I,(R+1):2R],1,mean))
win.data <- list(Y=Y,I=I,T=T)
```

```
################################################################################
# To run OpenBUGS  with AR model
################################################################################

output <- bugs(data = win.data,inits = NULL,model.file = "AR-1.txt",
parameters = c("sig2","beta.i","pi.1"), n.chains=1,n.iter=10000, n.burnin=8000,
debug=T,DIC=F,OpenBUGS.pgm=OpenBUGS.pgm,codaPkg=TRUE,WINE=WINE,WINEPATH=WIN
EPATH,useWINE=T)

################################################################################
# To read coda files
################################################################################

coda.output <- read.bugs(output)
beta.coda <- coda.output[,1:I]
pi.coda <- coda.output[,(I+1):((I+1+I)-1)]

################################################################################
# To compute tail probability of temporal dynamics
################################################################################

beta_mat <- as.matrix(beta.coda)
pi_mat <- as.matrix(pi.coda)

tocompute.pvals <- function(a){
 return((min(length(which(a <0 )),length(which(a > 0)))/2000 )*2)
                                                 }
# tail probability
b.pval <- apply(t(pi_mat) , MARGIN = 1, FUN = tocompute.pvals)
autoregressiveTable <- cbind(filtered.expr, b.pval)
write.table(autoregressiveTable,"/Users/sungheeoh/autoregressiveTableForD0VSD1.txt",row.n
ames=F, sep="\t")

################################################################################
# I-(2) Advanced AR Poisson model with MCMC algorithm in OpenBUGS(WinBUGS)
# To incorporate replicate factor and systematic artifact of lane effects
# To estimate W values from RUVSeq
################################################################################

sink("AR-2.txt")
cat("
model{

   for ( i in 1:I ) {
```

```
        for ( t in 1:T) {
            for(r in 1:R){ #replicates
                Y[i,r,t] ~ dpois( mu.y[i,r,t] )
                mu.y[i,r,t] <- exp( beta.i[i] + w.it[ i,r,t ] + W1[r,t] + W2[r,t])
                                        }
                                }
                        }
    #prior dist
    for ( i in 1:I ) {
        beta.i [i] ~ dnorm(0,1000.)
        pi.1[i] ~ dunif(-1., 1.)
                        }
    sig2 <- 1/tau2
    tau2 ~ dgamma(0.025, 0.025)
    for ( i in 1:I ) {
        for(r in 1:R){
            sig.w1[i,r] <- sig2/(1. - pi.1[i]*pi.1[i])
            w.it[i,r,1] ~ dnorm( 0.0, sig.w1[i,r])
                                }
        for (t in 2:T ) {
            for(r in 1:R){
                mean.wit[i,r,t] <- pi.1[i]*w.it[i,r,t-1 ]
                w.it[i,r,t] ~ dnorm (mean.wit[i,r,t] , sig2)
                                }
                        }
            }
}",fill=TRUE)
sink()

Y <-  array(dim=c(I,R,2))
Y[,,1] <- as.matrix(filtered.expr[1:I,1:R])
Y[,,2] <- as.matrix(filtered.expr[1:I,(R+1):(R+R)])
```

###
# To read systematic artifacts from RUVSeq
###

```
W <- read.table(paste(directory1,"W_VALUE_FOR EXPR.DATA.txt",sep=""), header=F,as.is=T)
W <- as.matrix(W)
W1 <- matrix(0,R,2)W2 <- matrix(0,R,2)
W1[1:R,1] <- W[1:R,1]
W1[1:R,2] <- W[(R+1):ncol(filtered.expr),1]
W2[1:R,1] <- W[1:R,2]
W2[1:R,2] <- W[(R+1):ncol(filtered.expr),2]
```

```
win.data <- list(Y=Y,I=I,T=T,R=R, W1=W1, W2=W2)

###########################################################################
# To run OpenBUGS
###########################################################################

output <- bugs(data=win.data, inits=NULL, model.file = "AR-2.txt", parameters =
c("sig2","beta.i","pi.1"),
n.chains=1,n.iter=10000,n.burnin=8000,debug=T,DIC=F,OpenBUGS.pgm=OpenBUGS.pgm,codaP
kg=TRUE,WINE=WINE,WINEPATH=WINEPATH,useWINE=T)

###########################################################################
# I-(3) Advanced AR model with MCMC algorithm in OpenBUGS(WinBUGS)
# To incorporate replicate factor and systematic artifact of lane effects
# To estimate W values from AR model
###########################################################################

sink("AR-3.txt")
cat("
model{

   for ( i in 1:I ) {
      for ( t in 1:T) {
         for(r in 1:R){ #replicates
            Y[i,r,t] ~ dpois( mu.y[i,r,t] )
            mu.y[i,r,t] <- exp( beta.i[i] + w.it[ i,r,t ] + W[r,t])
         }
      }
   }
   #prior dist
   for ( i in 1:I ) {
      beta.i [i] ~ dnorm(0,1000.)
      pi.1[i] ~ dunif(-1., 1.)
   }
   sig2 <- 1/tau2
   tau2 ~ dgamma(0.025, 0.025)
for(t in 1:T){
       for(r in 1:R){
                   W[r,t] ~ dnorm(0,1000)  #prior for W.
                            }
                   }
   for ( i in 1:I ) {
      for(r in 1:R){
```

```
          sig.w1[i,r] <- sig2/(1. - pi.1[i]*pi.1[i])
          w.it[i,r,1] ~ dnorm( 0.0, sig.w1[i,r])
       }
       for (t in 2:T ) {
          for(r in 1:R){
             mean.wit[i,r,t] <- pi.1[i]*w.it[i,r,t-1 ]
             w.it[i,r,t] ~ dnorm (mean.wit[i,r,t] , sig2)
                       }
                  }
               }
}",fill=TRUE)
sink()

   win.data <- list(Y=Y,I=I,T=T,R=R)

output <- bugs(data=win.data, inits=NULL, model.file = "AR-3.txt", parameters =
c("sig2","beta.i","pi.1"),
n.chains=1,n.iter=10000,n.burnin=8000,debug=T,DIC=F,OpenBUGS.pgm=OpenBUGS.pgm,codaP
kg=TRUE,WINE=WINE,WINEPATH=WINEPATH,useWINE=T)

###############################################################################
# I-(4) Advanced AR model with MCMC algorithm in OpenBUGS(WinBUGS)
# To incorporate replicate factor and systematic artifact of lane effects
# To estimate W values from AR model
# To allow varied numbers of replicates in each time point
# (Unbalanced design over a series of time points or when filtering some of samples)
#
# NOTE: Since our Bayesian AR model is implemented for repeatedly measured
# longitudinal time course RNA-Seq data over a series of time points, model update is
# based on paired previous expression levels (that is, current time point is estimated from the
# expression levels in previous time point), therefore, the number of replicates should be
# identical among groups of time points, if some of samples with bad quality in a group have
# been discarded, Please do filter out correspondingly repeatedly measured samples in the
# groups of other time points as well.
# For instance, in order to infer temporal patterns between D0 vs D7 w/o B29, B26 in D0
# should be also discarded to run AR-3 model, otherwise, if you want to keep the sample
# (B26), should run AR-1, not taking into account replicates, hence, we suggest the former
# case for unbalanced design and samples properly.
###############################################################################
```

< # II. Manual with an example data For Windows Users >

WINBUGS code

There are three steps for running the code - Model, Data and Initials.

Use the following steps to run his code:

1. From Menu Options, click "Model" and choose "Specification Tool".
2. Double-click (highlight) "model" in the code and click "check model" inside Specification Tool.
3. Click the left arrow under the Data step and double-click (highlight) "list and choose "load data" in Specification Tool.
4. Choose "compile" in the Specification Tool.
5. Click the left arrow under the Initial step and double-click (highlight) "list and choose "load inits" and then "gen Inits" in Specification Tool.
6. From Menu Options, click "Model" and choose "Updates...".
7. In Update Tool, set "8000" for updates and click "update" button. Wait until 'iteration" reaches 8000.
8. Next, from Menu Options, click "Inference" and choose "Samples...".
9. In Sample monitor Tool, write "beta.i" in node and hit "set" button, write "pi.1" and "set" and write "sig2" and hit "set".
10. Going back to Update Tool panel, set 2000 for "updates" and click "update" button. Wait until "iteration" reaches 10000.
11. In Sample Monitor Tool, type " * " in node window and hit "stats" to get summary statistics of model parameters (beta.i, pi.1 and sig2).
12. In Sample Monitor Tool, type " pi.1 " in node window and hit "coda" to get MCMC output of parameters, pi.1.

Equipment and equipment setup

- **Data:** Required expression data is a longitudinally measured time course RNA-Seq stimuli-response dataset with a baseline control group versus multiple later time points, allowing for replicates at each time point and multiple experimental factors.
- **Pre-required installation:** Wine and OpenBUGS (WinBUGS)
- **R** with the latest version

Hardware: Mac OS X Sierra version 10.12.6; 8 GB (equivalent or later version and larger size of RAM) and Windows 7 using Parallels Desktop in Mac Pro, 3.5 GHz, 64 GB memory (equivalent or later version and larger size of RAM).

Required data: These analytical pipelines are illustrated with an example of our longitudinal stimuli-response time course data in the Bos_taurus genome to demonstrate how to address systematic artifacts and isoform diversity and how to precisely analyze temporal dynamics with multiple factors for RNA-Seq data. In this example, we are simply comparing the control baseline time point to later time points. However, of course, our proposed method can be directly applied for a larger set with multiple time points (longer than two time points), and it can be appropriately applied for longitudinal time course data with more than two time points.

Hardware setup: To run dynamic AR models in OpenBUGS (WinBUGS) with MCMC simulations and to process RNA-Seq data with multiple replicates and factors, the machine used for these pipelines is recommended to have at least 8 GB (or more) of RAM. We recommend using OpenBUGS. It is more efficient and faster than WinBUGS for the computational time.

Downloading sequencing data and organizing required data: As a preprocessing procedure, generate bam or sam files, quantify expression levels using the Tuxedo method and prepare the settings of "expression data", "parameter settings", and "initial values" for the defined Bayesian dynamic AR model, as shown in Manual. For initial values, we recommend using the generator of initial values in either OpenBUGS or WinBUGS.

Timing

- **When ignoring the variability of replicates by taking the average expression level value for each group of pooled data**

~2 hours to run the dynamic AR model with MCMC 10,000 iterations with 8,000 burn-ins and to write down coda out files of simulations under the current Mac OS X settings and our exemplified data set. ~ less than 1 hour to run the dynamic AR model with MCMC 10,000 iterations with 8,000 burn-ins and to write down coda out files of simulations under the current Windows 7 using Parallels Desktop in Mac Pro 3.5 GHz and 64 GB memory (equivalent or later version and larger size of RAM) setting. Computational times might vary depending on the size of samples for a given dataset and equipment settings.

- **When ignoring the variability of replicates by taking the average expression level value within each group of unpooled data**

~2 hours under the identical settings on Mac OS X Sierra for a given dataset and equipment. (~ less than 1 hour on Windows).

- **When accounting for the variability of replicates in the Poisson gamma model on pooled data**

~4 hours under the identical settings for equipment when filtering out genes with zero and low expression, resulting in ~ 10,000 genes (isoforms) (~ 1 hour on Windows)

- **When accounting for the variability of replicates in the Poisson gamma model on pooled data after correction**

~4 hours under the identical settings for equipment when filtering out genes with zero and low expression, resulting in ~ 10,000 genes (isoforms) (~ 1 hour on Windows).

Limitations

This study is not a comprehensive comparative study to rank which methods are better in terms of the performance of true discovery rates and the power of detection due to a variety of scenarios in multiple synthetic datasets, various real data applications, different differential expression methods and other downstream analyses. In contrast, our study is focused on emphasizing that more precise analytical strategies are necessary to address complicated experiments, such as time course data with stimulated responses and combined multiple heterogeneous datasets (across different platforms, laboratories, sequencing dates, machines, and investigators). Additionally, it is not a simulation study where we know which genes and isoforms are differentially and equally expressed, and we could not compute the extent of decreased sensitivity and specificity for each comparative test. However, we confirmed our findings detected by proposed methods in pipelines by comparing to known genes detected by static method in the previous study (Baldwin et al., 2012) and also, by qPCR validation for the selected gold-standard gene list.

Supplementary Figures

Supplementary Figure 1-(1). The analytical pipelines performed in this study.

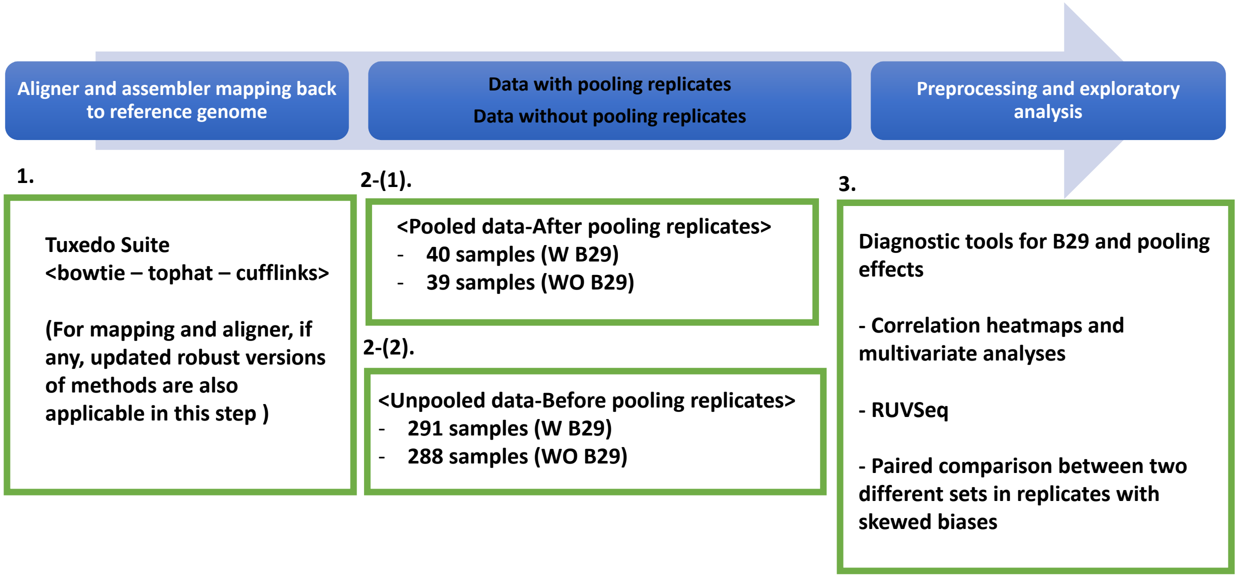

Supplementary Figure 1-(2). The analytical pipelines performed in this study.

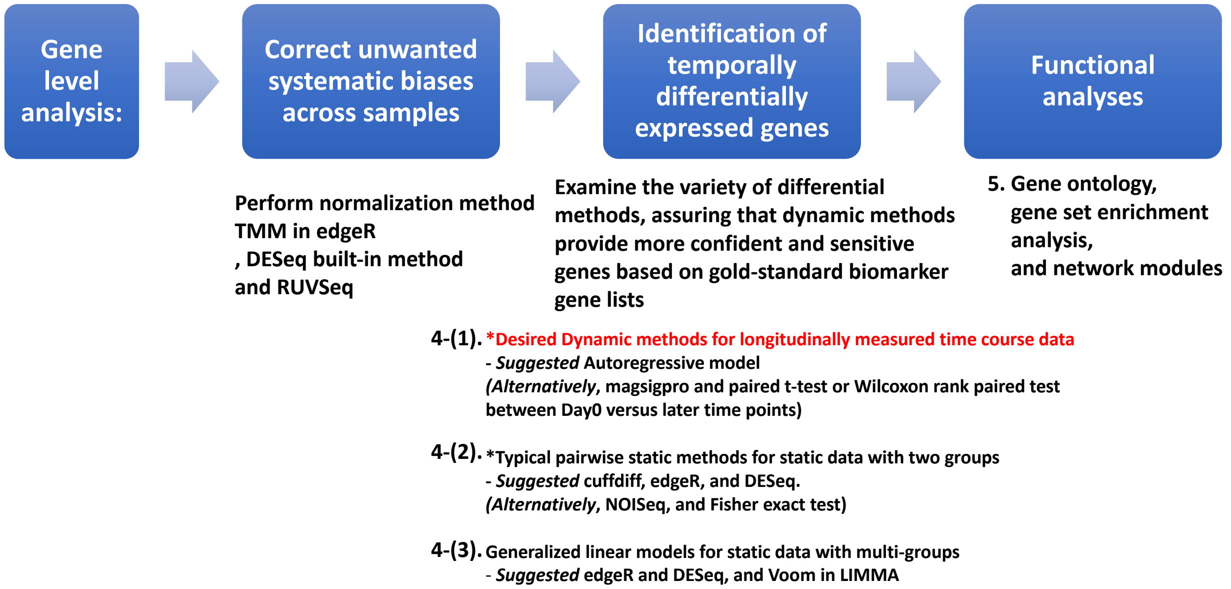

Supplementary Figure 1-(3). The analytical pipelines performed in this study.

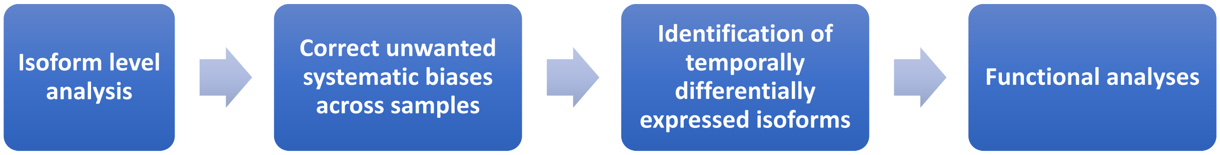

Perform normalization method TMM in edgeR , DESeq built-in method and RUVSeq

Before correction,
6-(1). Suggested cuffdiff and DEXSeq

After correction,
6-(2). Suggested dynamic autoregressive model on the quantification of transcripts and exon levels for dynamic data
6-(3). Suggested edgeR and DESeq on the quantification of transcripts and exon levels for static data

5. Gene ontology, gene set enrichment analysis, and network modules

Supplementary Figure 1-(4). The analytical pipelines performed in this study.

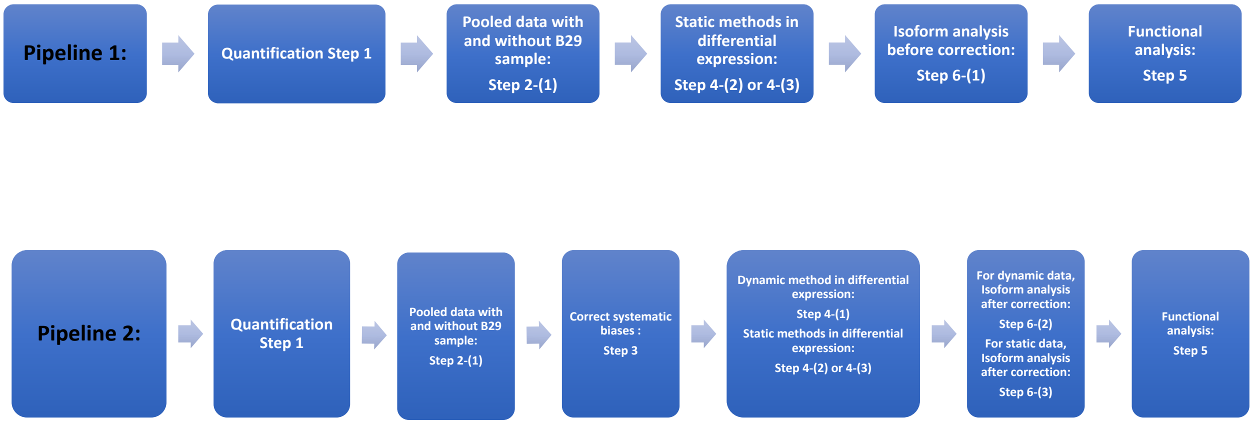

Supplementary Figure 1-(5). The analytical pipelines performed in this study.

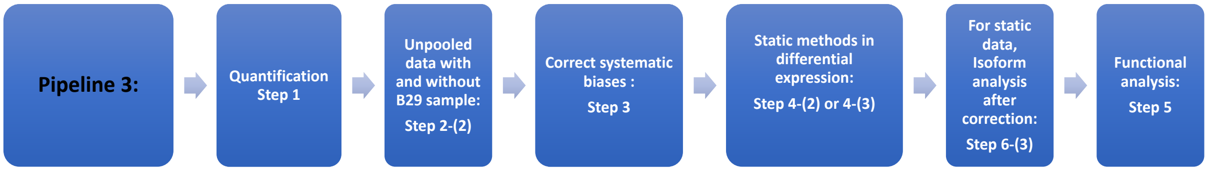

Supplementary Figure 2-(1). Comparison of inter-pipelines between after and before correction of unwanted biases (and also, between inclusion and exclusion of sample B29) when comparing D0 versus D7 at FDR=0.1 for after-pooling samples for static methods (edgeR and DESeq), respectively.

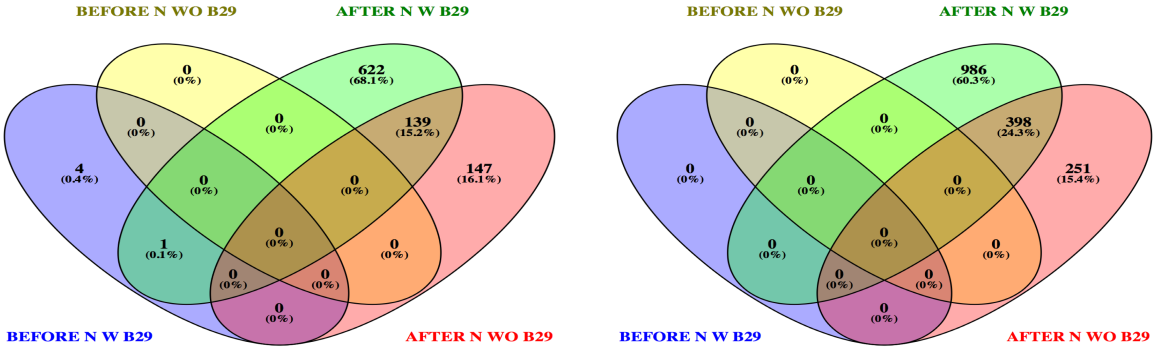

Note: In labels, "After" and "before" represent datasets, after- and before-correction, respectively. And "W" and "WO" represent including and filtering out B29 sample in Day 7 samples, respectively. And, for "BEFORE N WO B29", there are no significant genes.

Supplementary Figure 2-(2). Comparison of intra-pipeline before correction of unwanted biases when comparing D0 versus later time points at cutoff=0.1 for after-pooling samples for Bayesian dynamic AR model of genes with and without B29, respectively. And also, the given table shows the intersection of temporally differential expression between comparisons.

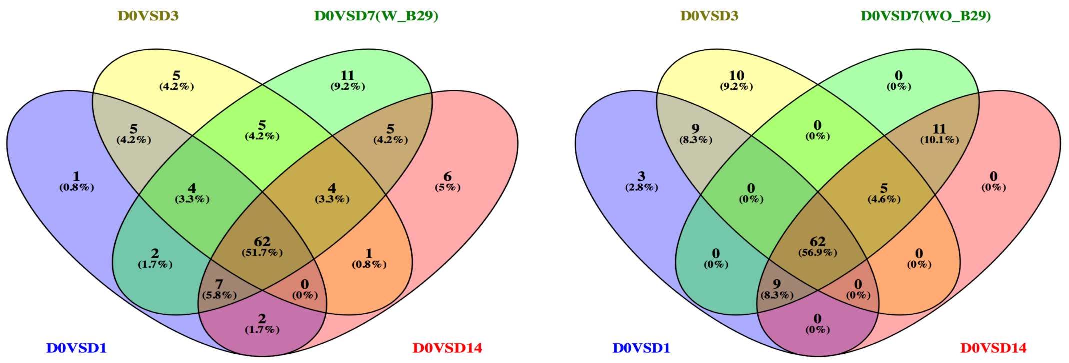

D1 D3<br>(83) (86)	D3 D7<br>(86) (100)	D7 D14<br>(100) (87)
71	75	78

D1 D3<br>(83) (86)	D3 D7<br>(86) (87)	D7 D14<br>(87) (87)
71	67	87

Supplementary Figure 2-(3). Comparison of inter-pipelines between before and after correction of unwanted biases for D0 versus D1 group at the tail probability of 0.1 after pooling samples for Bayesian dynamic AR method, where (after) and (before) represent "after and before correction of unwanted biases", respectively.

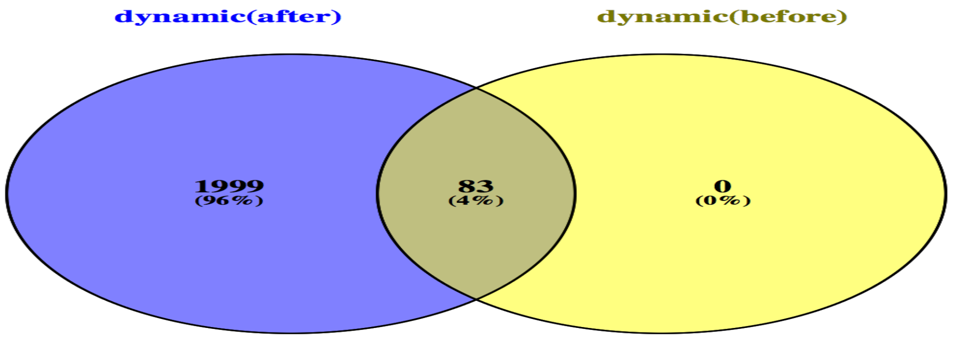

Supplementary Figure 2-(4). Comparison of inter-pipelines between before and after correction of unwanted biases for D0 versus D3 group at the tail probability of 0.1 after pooling samples for Bayesian dynamic AR method, where (after) and (before) represent "after and before correction of unwanted biases", respectively.

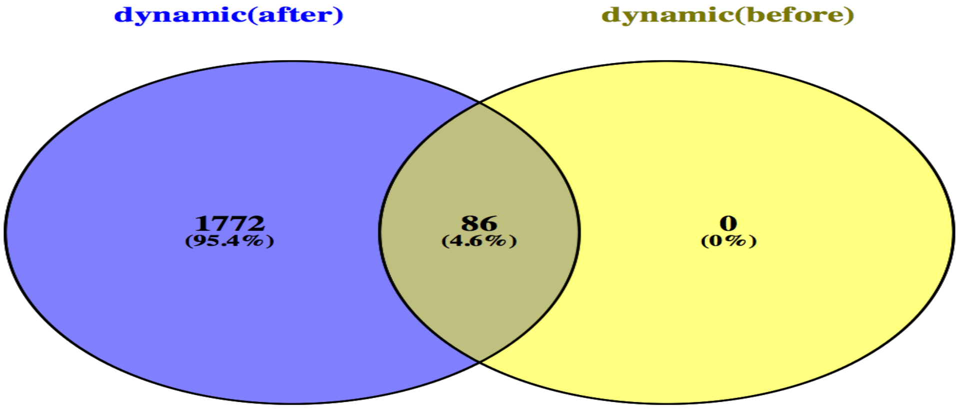

Supplementary Figure 2-(5). Comparison of inter-pipelines between before and after correction of unwanted biases for D0 versus D7 group (with B29 sample) at FDR=0.1 after pooling samples for Bayesian dynamic AR method.

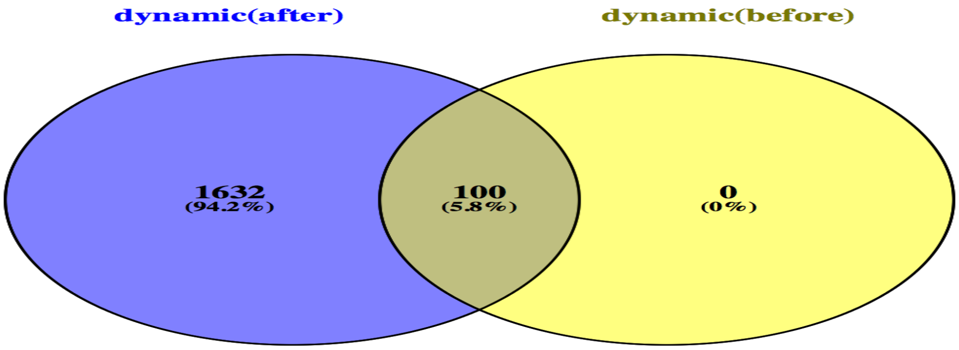

Supplementary Figure 2-(6). Comparison of inter-pipelines between before and after correction of unwanted biases for D0 versus D7 group (without B29 sample) at the tail probability of 0.1 after pooling samples for Bayesian dynamic AR method.

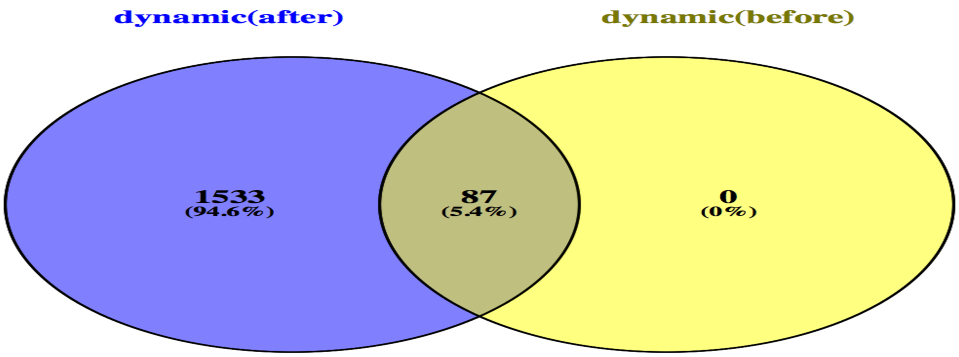

Supplementary Figure 2-(7). Comparison of inter-pipelines between before and after correction of unwanted biases and discarding B29 sample for D0 versus D7 group at the tail probability of 0.1 after pooling samples for Bayesian dynamic AR method, where (A) and (B) represent "After and Before correction of unwanted biases", respectively, and also, W and WO represent "with and without B29 sample in D7 group", respectively.

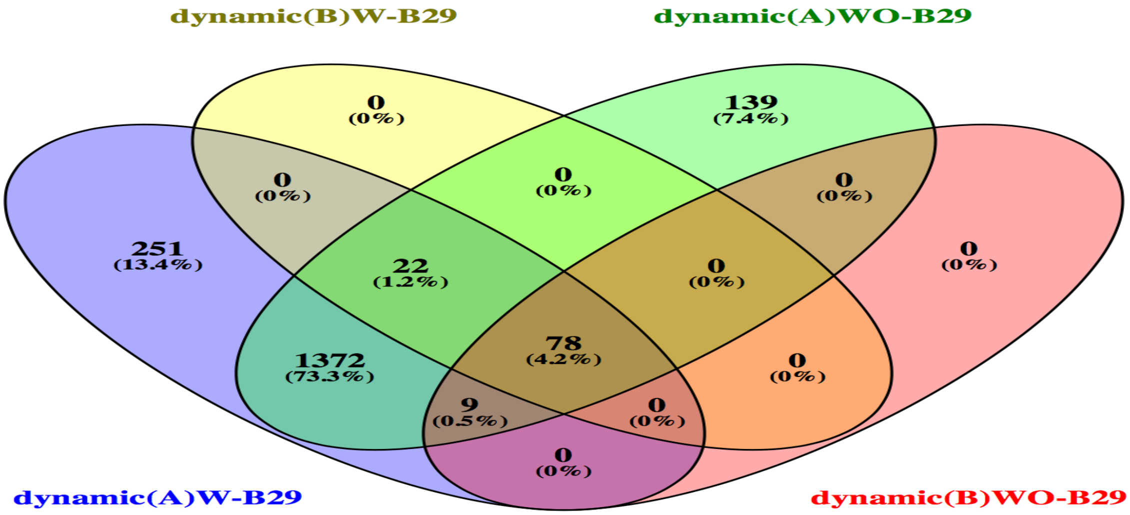

Supplementary Figure 2-(8). Comparison of inter-pipelines between before and after correction of unwanted biases for D0 versus D14 group at the tail probability of 0.1 after pooling samples for Bayesian dynamic AR method.

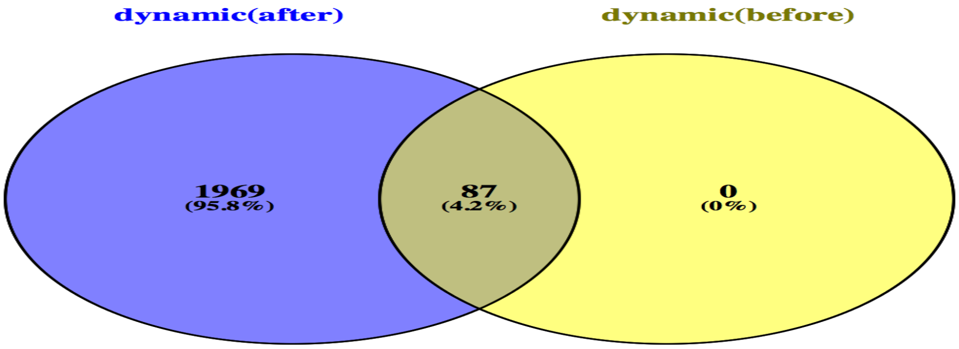

Supplementary Figure 2-(9). Comparison between after correction of unwanted biases for D0 versus D1 to D14 at the tail probability of 0.1 after pooling samples for Bayesian dynamic AR method. It is the comparison of intra-pipeline for D0 versus D1 to D14 samples after pooling and after correction, where D7 group excludes B29 sample and (A) represents "after correction of unwanted biases".

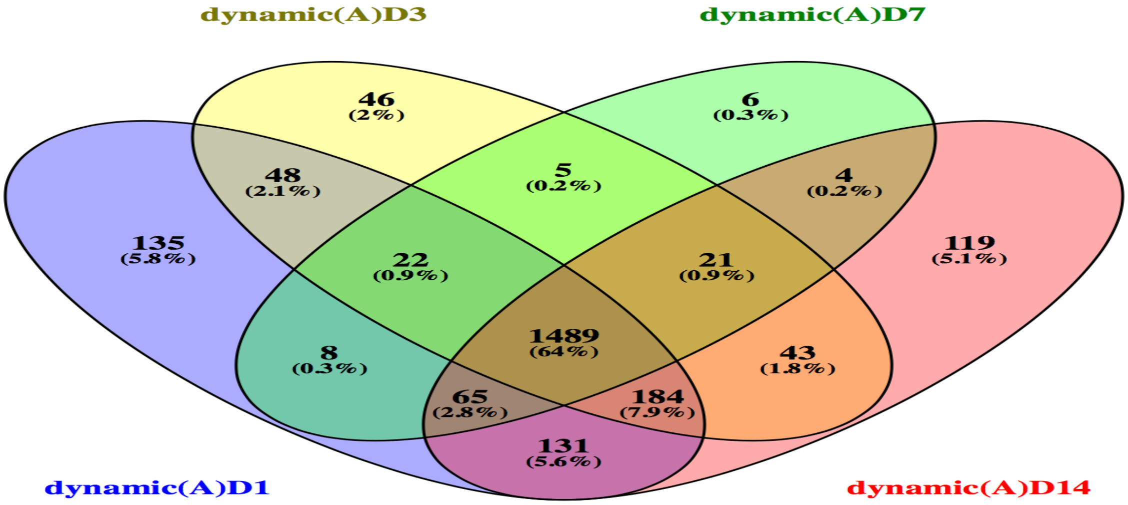

Supplementary Figure 2-(10). Comparison between after correction of unwanted biases for D0 versus D1 to D14 at the tail probability of 0.1 after pooling samples for Bayesian dynamic AR method. It is the comparison of intra-pipeline for of D0 versus D1 to D14 samples after pooling and before correction, where D7 group excludes B29 sample and (B) represents "before correction of unwanted biases".

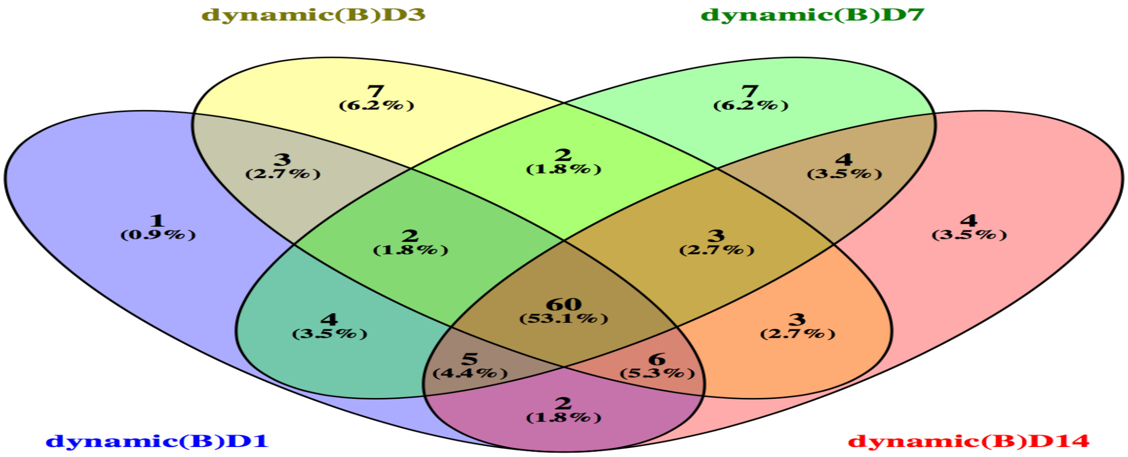

Supplementary Figure 2-(11). Comparison between after correction of unwanted biases for D0 versus D1 to D14 at FDR=0.1 after pooling samples for static edgeR method. It is the comparison of intra-pipeline for D0 versus D1 to D14 samples after pooling and after correction, where D7 group excludes B29 sample.

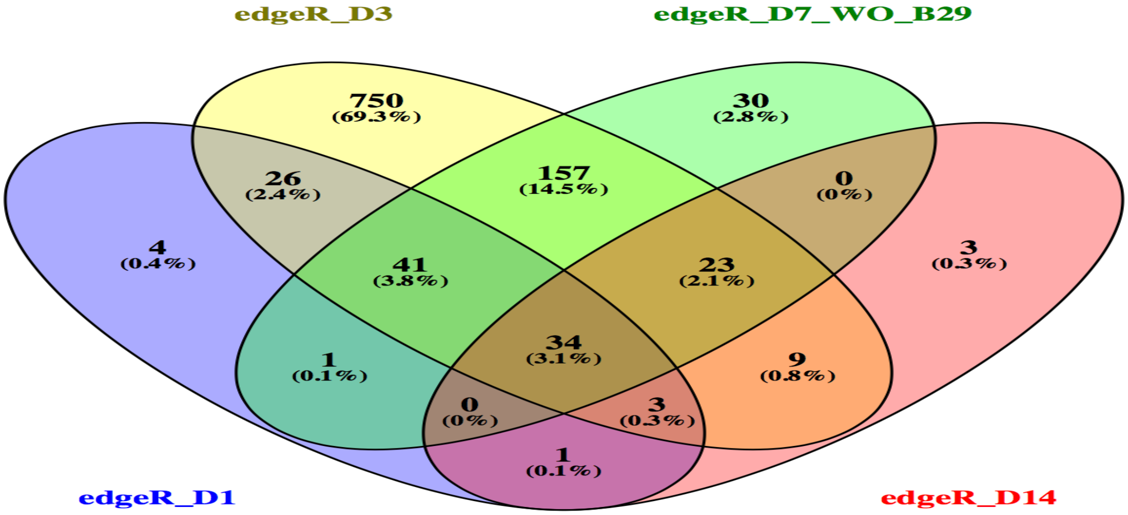

Supplementary Figure 2-(12). Comparison between after correction of unwanted biases for D0 versus D1 to D14 at FDR=0.1 after pooling samples for static edgeR method. It is the intra-comparison of D0 versus D1 to D14 samples after pooling and after correction, where D7 group includes B29 sample.

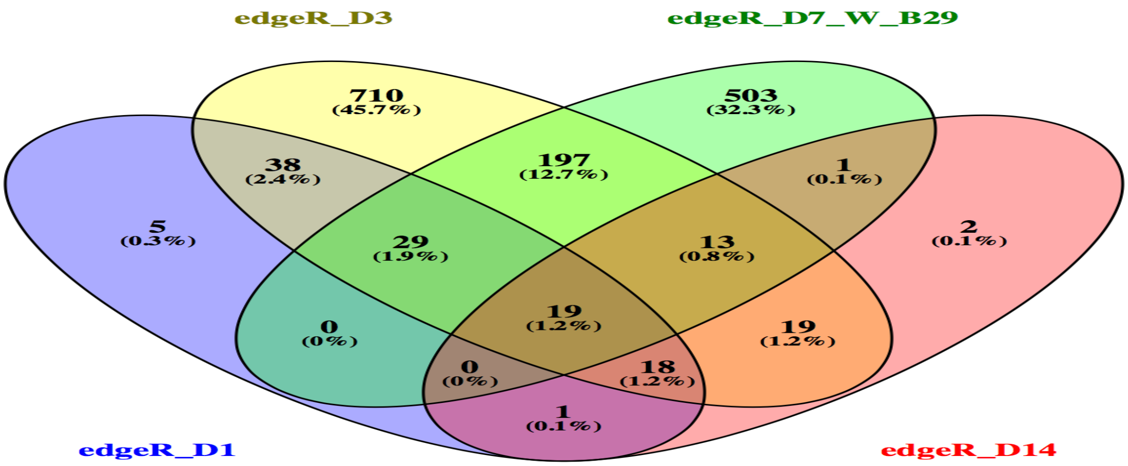

Supplementary Figure 2-(13). Comparison between before correction of unwanted biases for D0 versus D1 to D14 at FDR=0.1 after pooling samples for static edgeR method. It is the comparison of intra-pipeline for D0 versus D1 to D14 samples after pooling and before correction, where D7 group includes B29 sample.

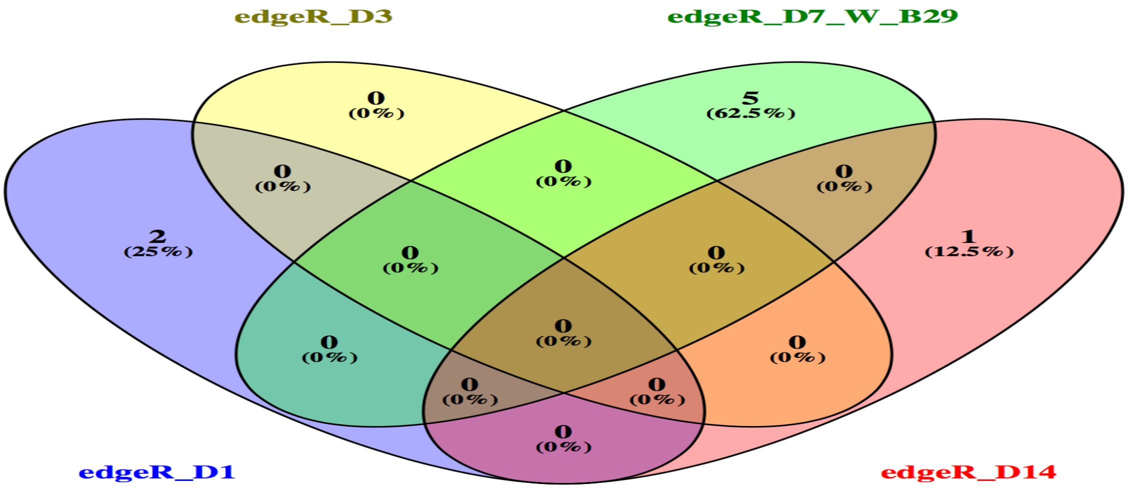

Supplementary Figure 2-(14). Comparison between before correction of unwanted biases for D0 versus D1 to D14 at FDR=0.1 after pooling samples for static edgeR method. It is the comparison of inter-pipelines for D0 versus D7 samples after pooling and after/before correction, where (A) and (B) represent "after and before correction of unwanted biases" and W and WO represent with and without B29 sample in D7 group.

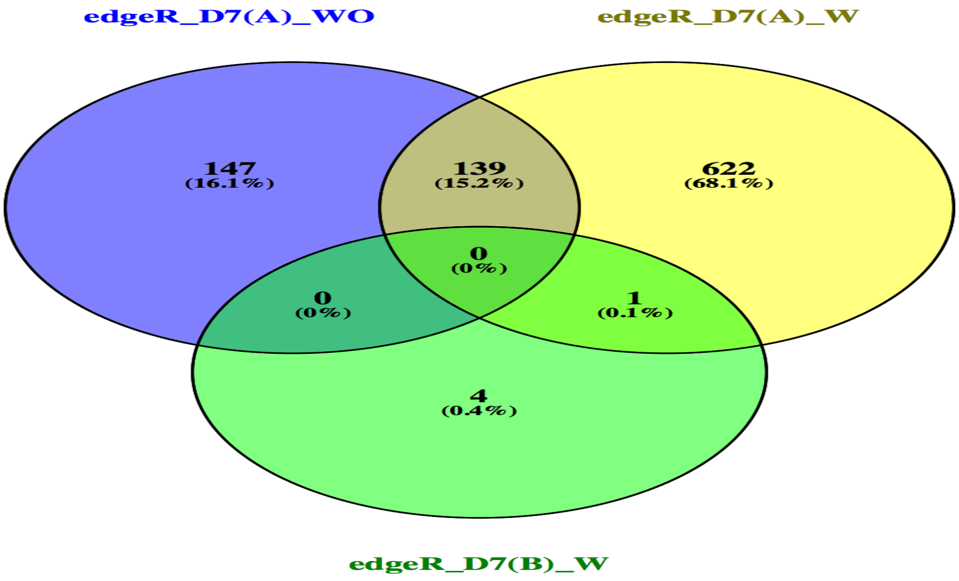

Supplementary Figure 3-(1). A heatmap of 147 genes (correction and filtering specific genes) detected by edgeR after correction of biases and without sample B29.

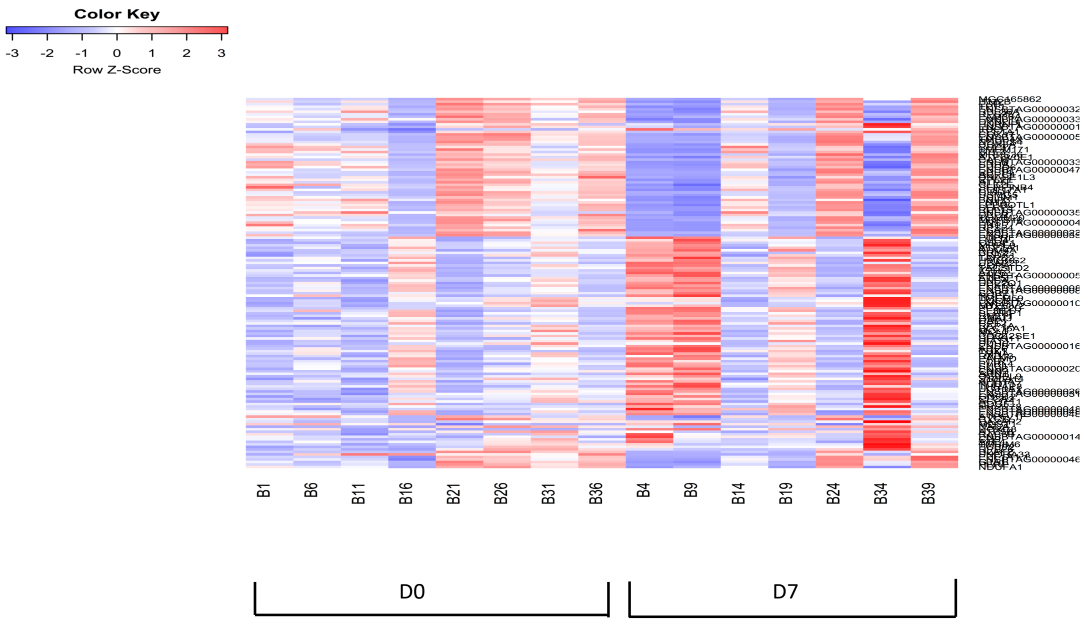

Supplementary Figure 3-(2). A heatmap of 251 genes (correction and filtering specific genes) detected by DESeq after correction of biases and with sample B29.

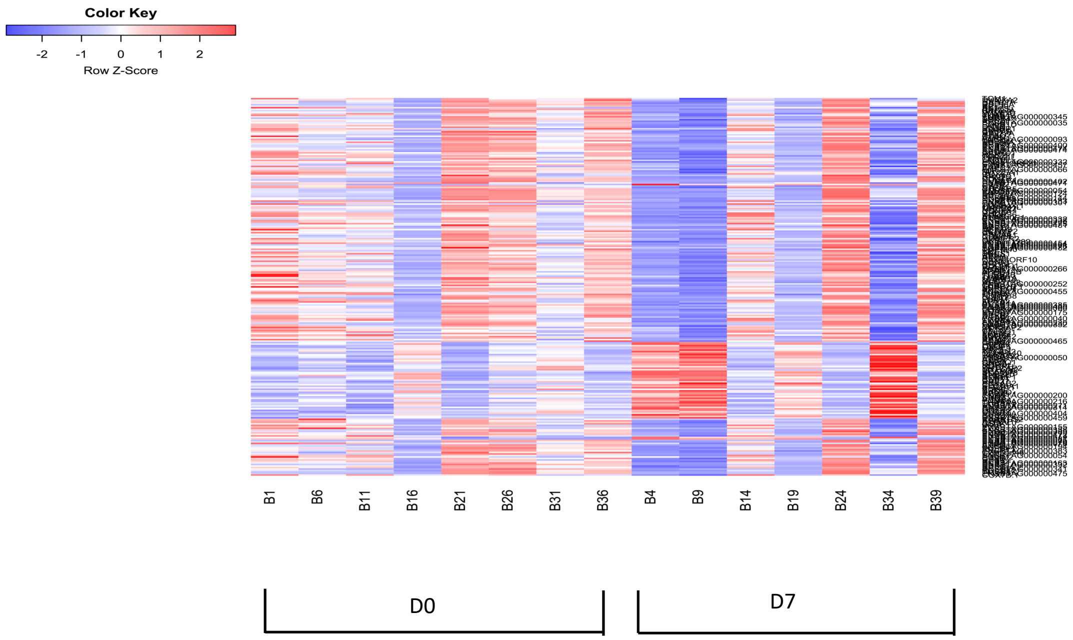

Supplementary Figure 3-(3). A heatmap of 100 and 87 genes detected by Bayesian dynamic AR method before correction of biases, with and without sample B29, respectively.

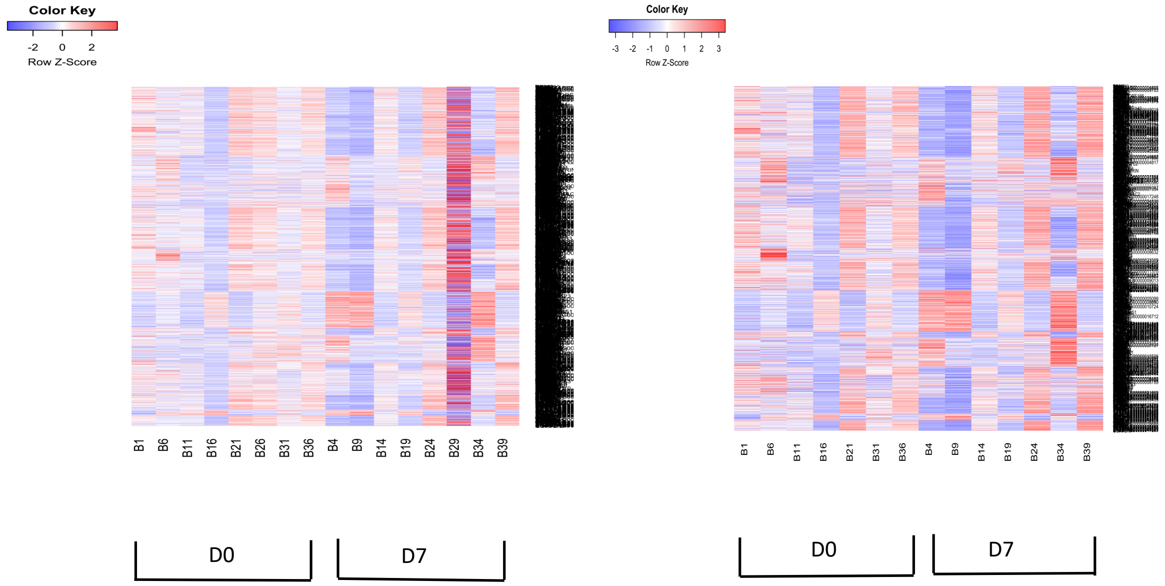

Supplementary Figure 3-(4). A heatmap of 1620 genes detected by Bayesian dynamic AR method on pooled data after correction.

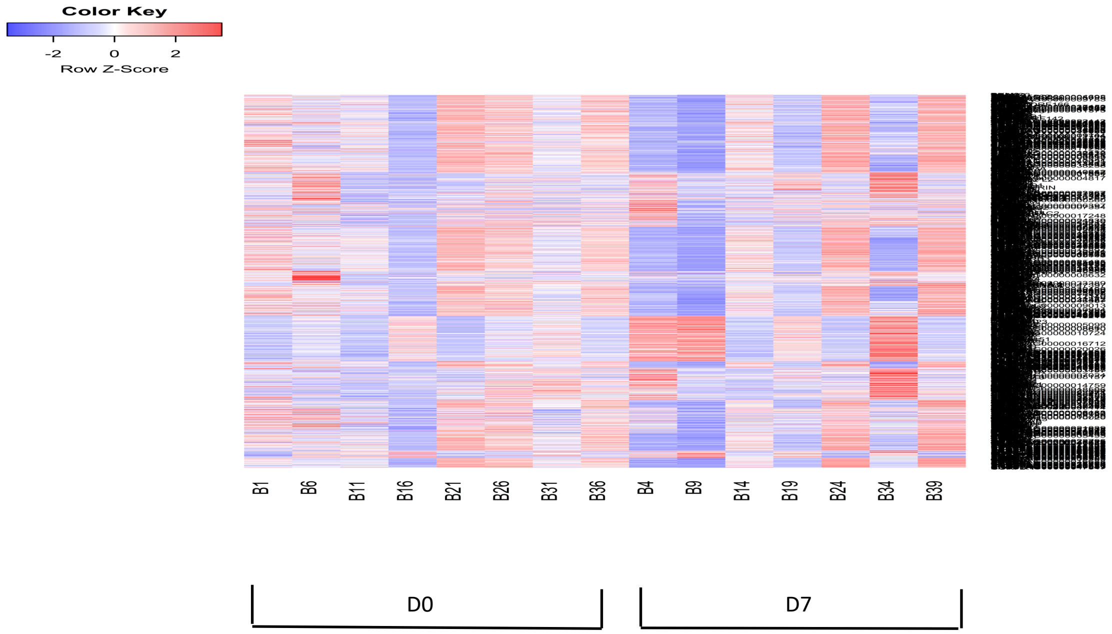

Supplementary Figure 4. Correlation heatmaps with and without sample B29 (291 and 288 samples on before pooling data) of genes, respectively. (Upper panel) PCA plots before and after correction of systematic biases with and without sample B29 of genes, respectively. (Lower panel)

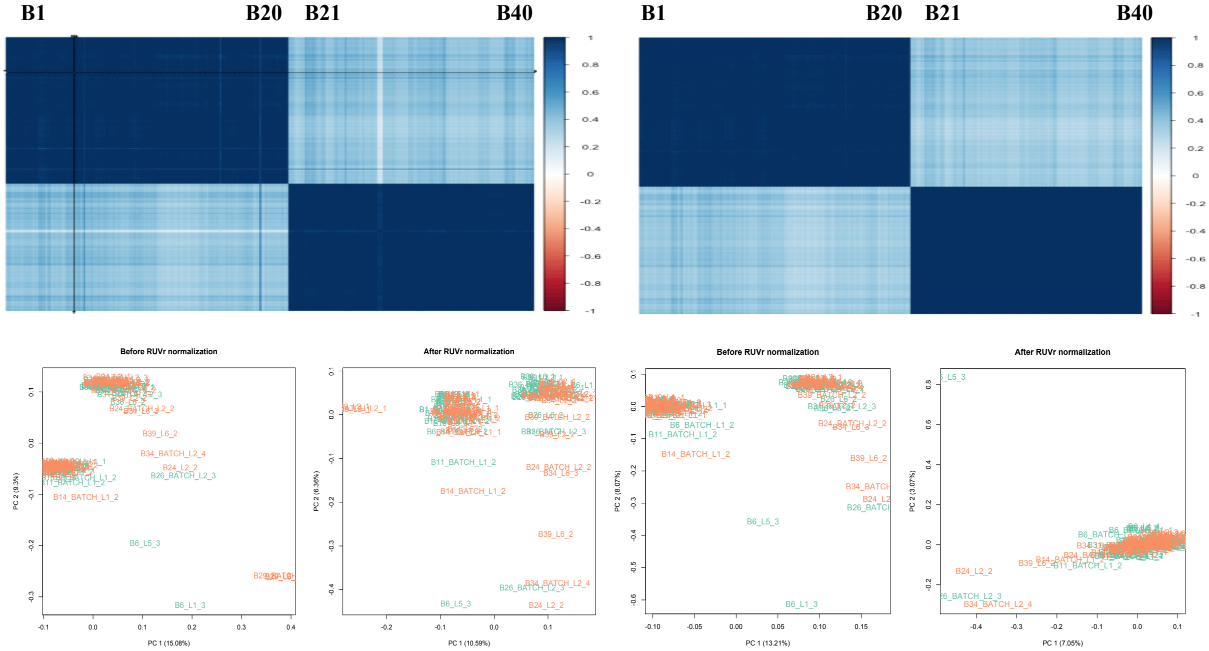

Supplementary Figure 5. Comparison of inter-pipelines between before and after correction of unwanted biases and discarding sample B29 when comparing D0 versus D7 at FDR=0.1 before pooling samples for static method (edgeR).

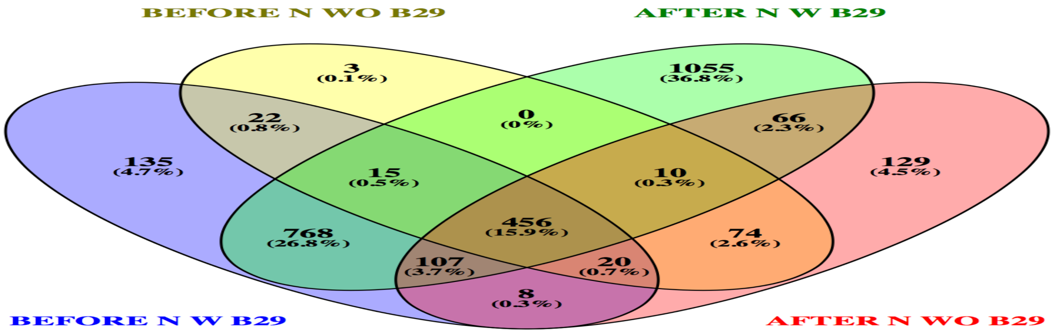

Note: In labels, "After" and "before" represent datasets, after- and before-correction, respectively. And "W" and "WO" represent including and filtering out B29 sample in Day 7 samples, respectively.

Supplementary Figure 6-(1). Network modules in protein-protein interactions for significantly differentially expressed genes detected by the static edgeR method from D0 versus D1

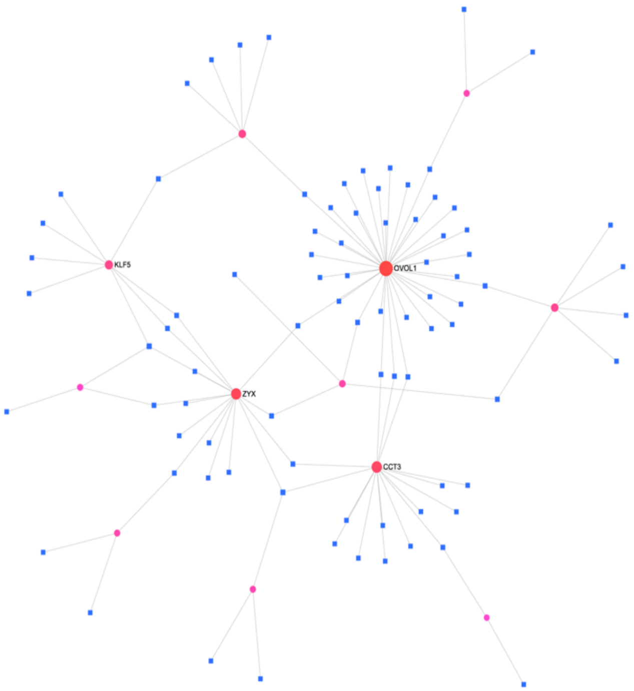

Supplementary Figure 6-(2). Network modules in gene and miRNA interactions for significantly differentially expressed genes detected by the static edgeR method from D0 versus D14.

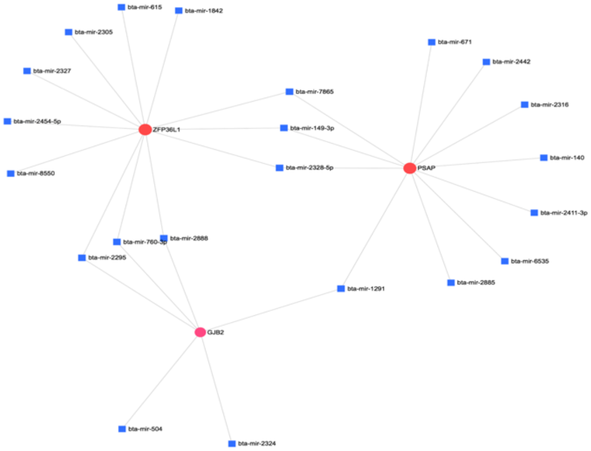

Supplementary Figure 6-(3). Network modules in protein-protein interactions for significantly differentially expressed genes detected by the static edgeR method from D0 versus D1 through D7 without B29 sample.

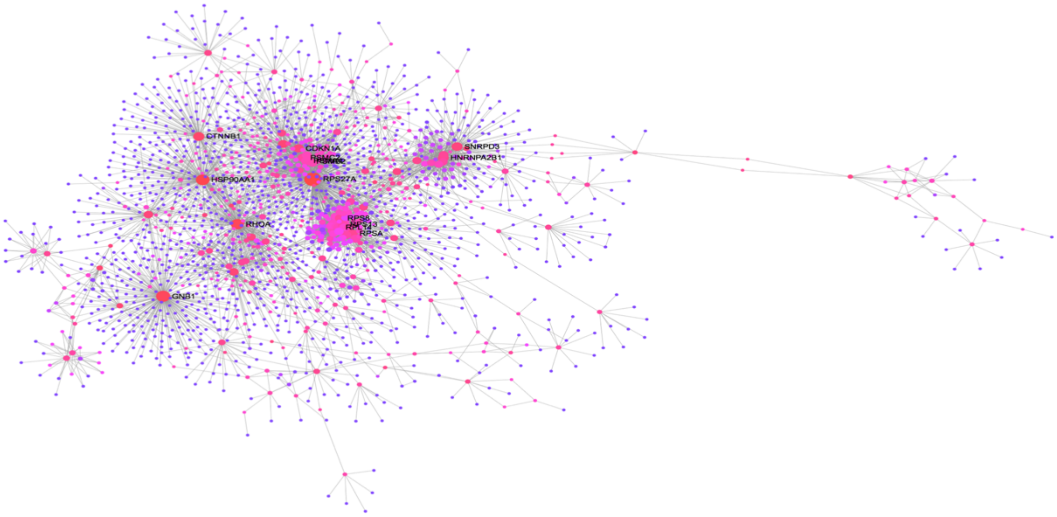

Supplementary Figure 6-(4). Network modules in gene and miRNA interactions for significantly differentially expressed genes detected by the static edgeR method from D0 versus D1 versus D7 without B29 sample.

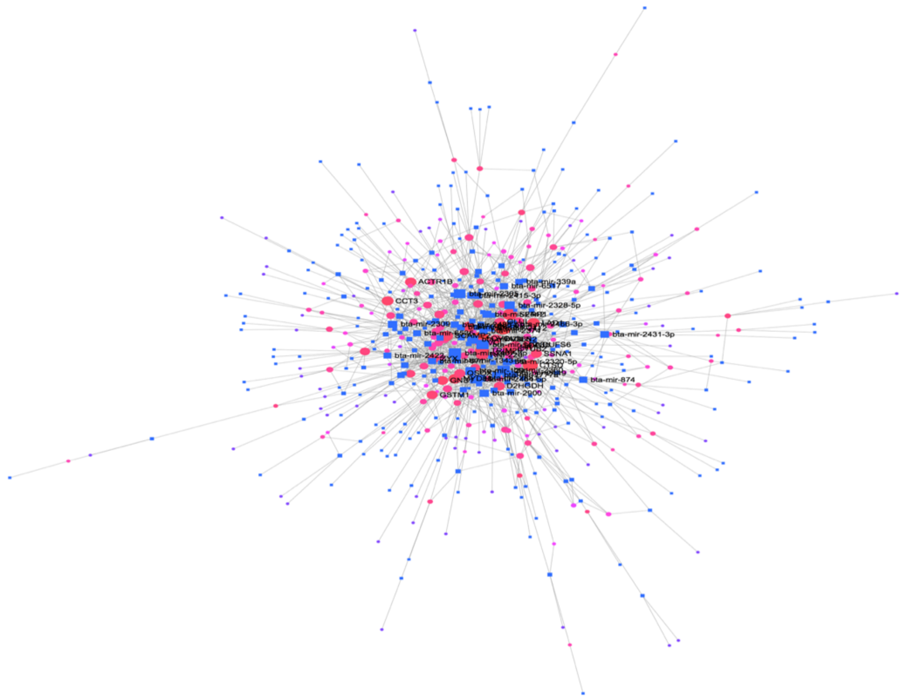

Supplementary Figure 7-(1). Network modules in protein-protein interactions for significantly differentially expressed genes detected by the Bayesian dynamic AR method from D0 versus D1.

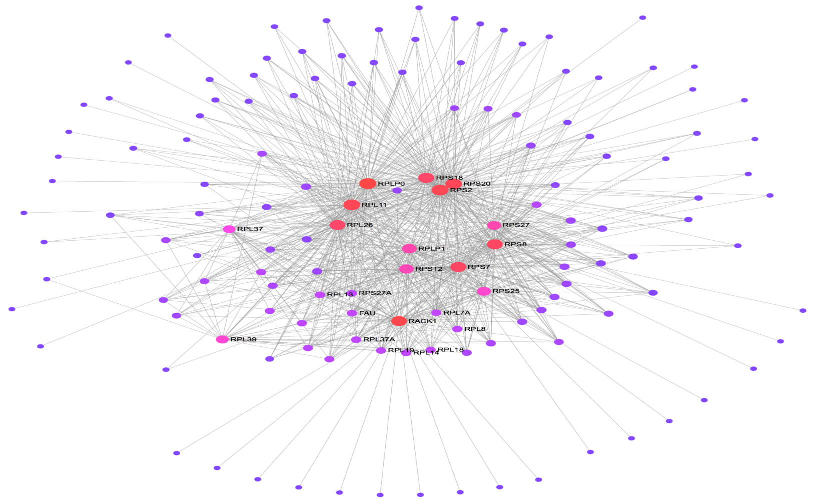

Supplementary Figure 7-(2). Network modules in gene and miRNA interactions for significantly differentially expressed genes detected by the Bayesian dynamic AR method from D0 versus D1.

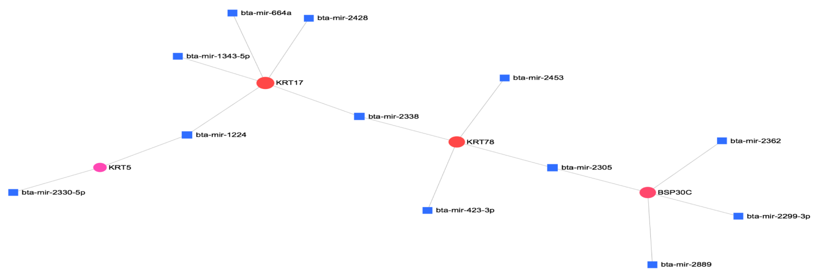

Supplementary Figure 7-(3). Network modules in protein-protein interactions for significantly differentially expressed genes detected by the Bayesian dynamic AR method from D0 versus D3.

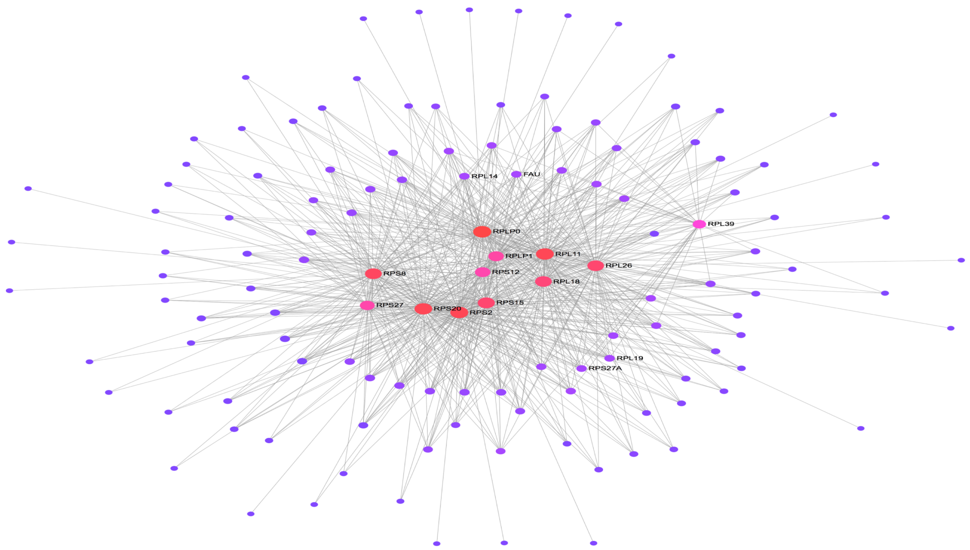

Supplementary Figure 7-(4). Network modules in gene and miRNA interactions for significantly differentially expressed genes detected by the Bayesian dynamic AR method from D0 versus D3.

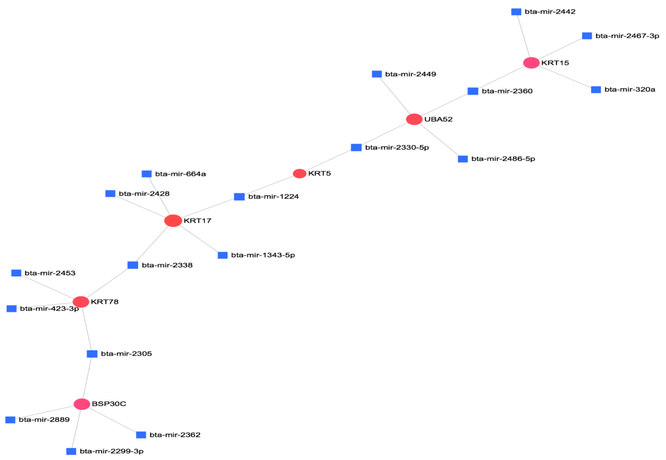

Supplementary Figure 7-(5). Network modules in protein-protein interactions for significantly differentially expressed genes detected by the Bayesian dynamic AR method from D0 versus D7 without B29 sample.

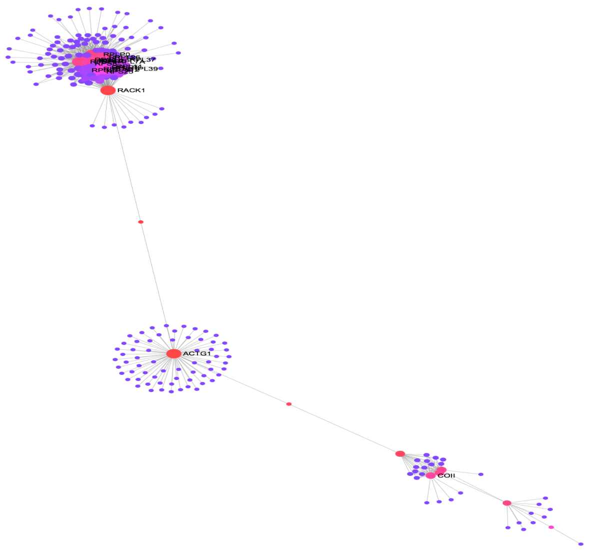

Supplementary Figure 7-(6). Network modules in gene and miRNA interactions for significantly differentially expressed genes detected by the dynamic AR method from D0 versus D7 without B29 sample.

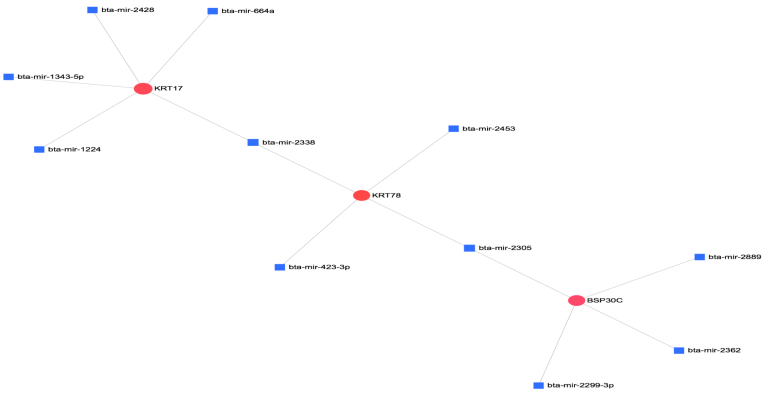

Supplementary Figure 7-(7). Network modules in protein-protein interactions for significantly differentially expressed genes detected by the Bayesian dynamic AR method from D0 versus D14.

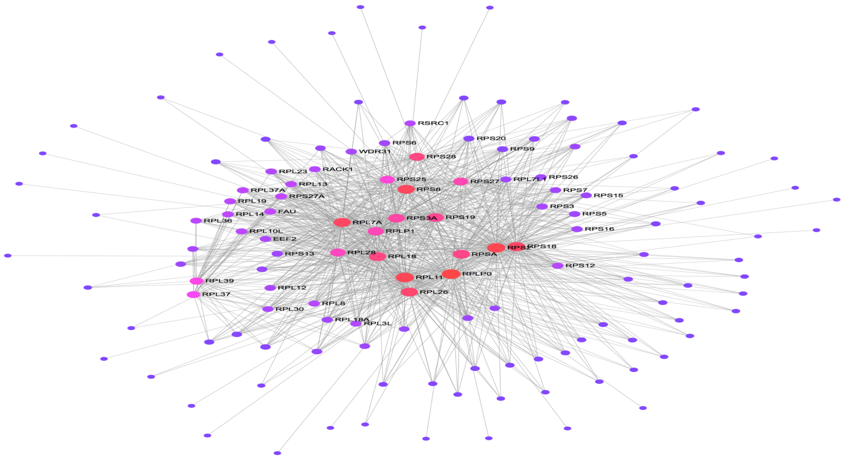

Supplementary Figure 7-(8). Network modules in gene and miRNA interactions for significantly differentially expressed genes detected by the Bayesian dynamic AR method from D0 versus D14.

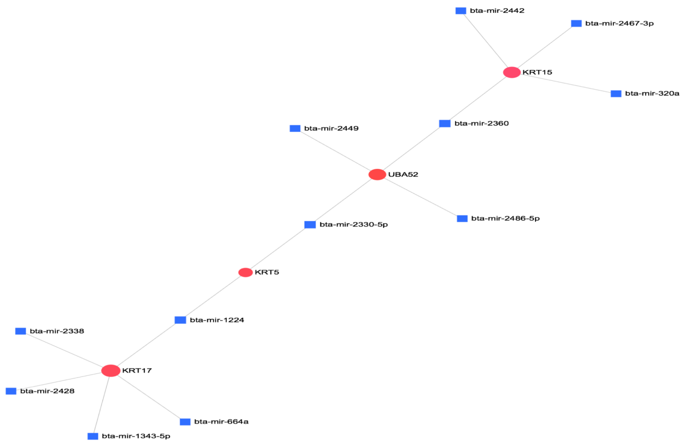

SUPPLEMENTARY MOVIE LEGENDS

Supplementary Movie 1-(1). It represents 3D PCA plot of gene expression data with B29 sample in D7 group after pooling replicates and before correction.
Supplementary Movie 1-(2). It represents 3D PCA plot of gene expression data without B29 sample in D7 group after pooling replicates and before correction.

Supplementary Movie 2-(1). It represents 3D PCA plot of genes with B29 sample before pooling replicates before correction.
Supplementary Movie 2-(2). It represents 3D PCA plot of genes without B29 sample, respectively before pooling replicates before correction.

TABLE LEGENDS

Data S1. Detailed information of sample profile

Data S2. Correlation Heatmaps for after and before pooling data

Data S3-(1). Significant genes in D0 versus D1 (to D14) with FDR 0.1 after pooling and before correction
Data S3-(2). Significant genes in D0 versus D1 (to D14) with FDR 0.1 after pooling and after correction
Data S3-(3). Significant genes in D0 versus D1 (to D14) with FDR 0.1 before pooling and before correction
Data S3-(4). Significant genes in D0 versus D1 (to D14) with FDR 0.1 before pooling and after correction

Data S4-(1). Significant genes in D0 versus D1 (to D14) with the tail probability of 0.1 after pooling and before correction using Bayesian dynamic AR method
Data S4-(2). Significant genes in D0 versus D1 (to D14) with the tail probability of 0.1 after pooling and with replicates using Bayesian dynamic AR method
Data S4-(3). Significant genes in D0 versus D1 (to D14) with the tail probability of 0.1 after pooling and after correction for replicates and lane effect using Bayesian dynamic AR method
Data S4-(4). Significant genes in D0 versus D1 (to D14) with the tail probability of 0.1 before pooling and before correction using Bayesian dynamic AR method

Data S5. Functional analysis after pooling and after correction using edgeR static and Bayesian dynamic AR method

Data S6-(1). Isoform diversity in D0 versus D1 (to D14) with FDR 0.1 before pooling and before correction
Data S6-(2). Isoform diversity in D0 versus D1 (to D14) with FDR 0.1 before pooling and after correction

Data S7. qRT PCR validation for the selected gold-standard gene list that has been detected by our proposed dynamic method, but not by other static methods
