# Supplementary figures and images for: Temporal dynamics in meta longitudinal RNA-Seq data
Source: Sci Rep. 2019 Jan 24;9:763. doi: 10.1038/s41598-018-37397-7 (PMC6345883; doi:10.1038/s41598-018-37397-7)

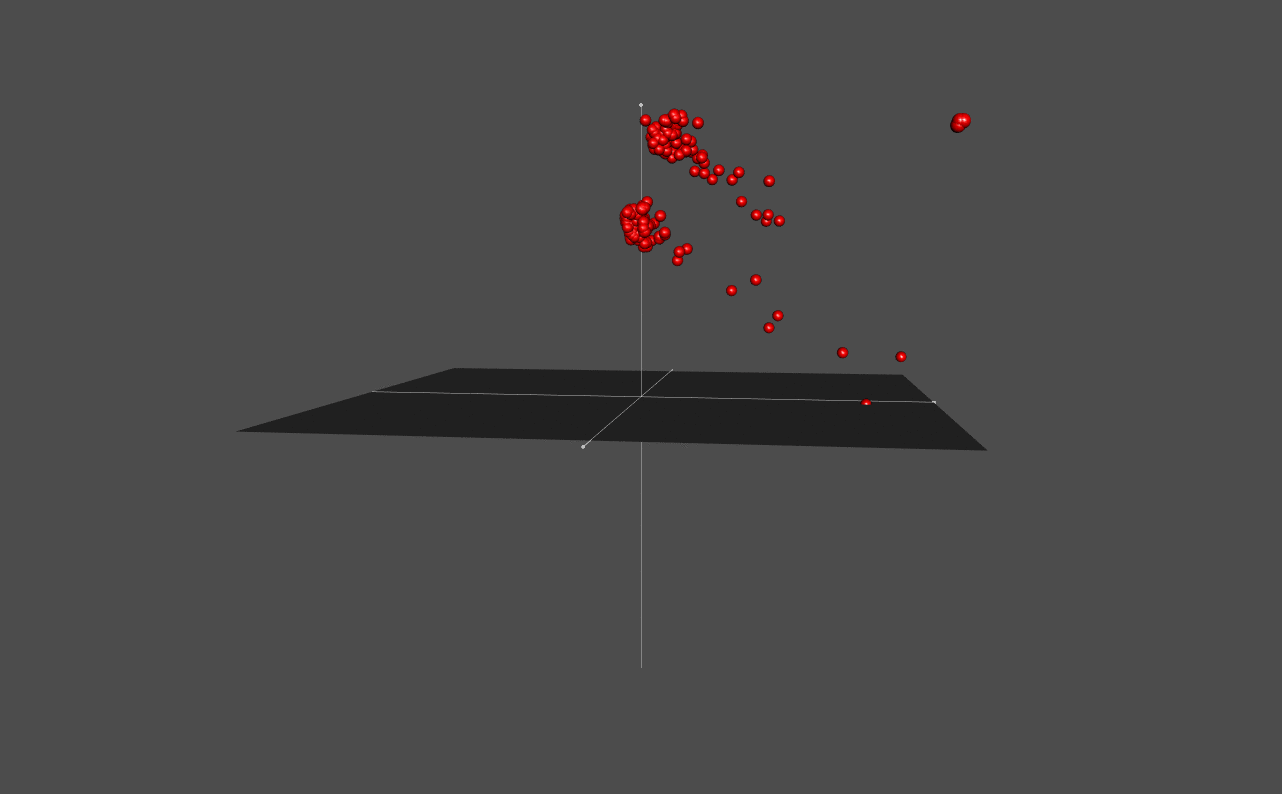

Supplement: Supplementary file 16 — Supplemental Vide S1-(1) [file 41598_2018_37397_MOESM16_ESM.gif]

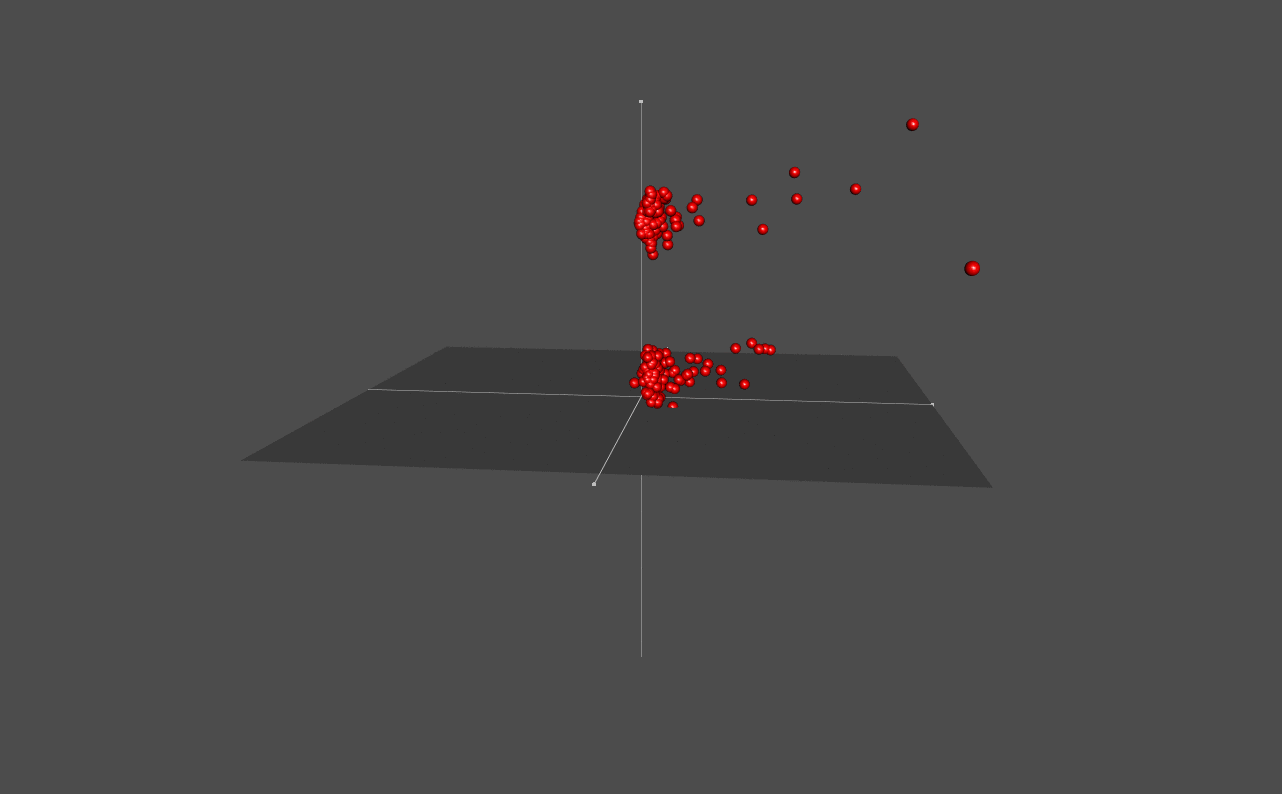

Supplement: Supplementary file 17 — Supplemental Vide S1-(2) [file 41598_2018_37397_MOESM17_ESM.gif]

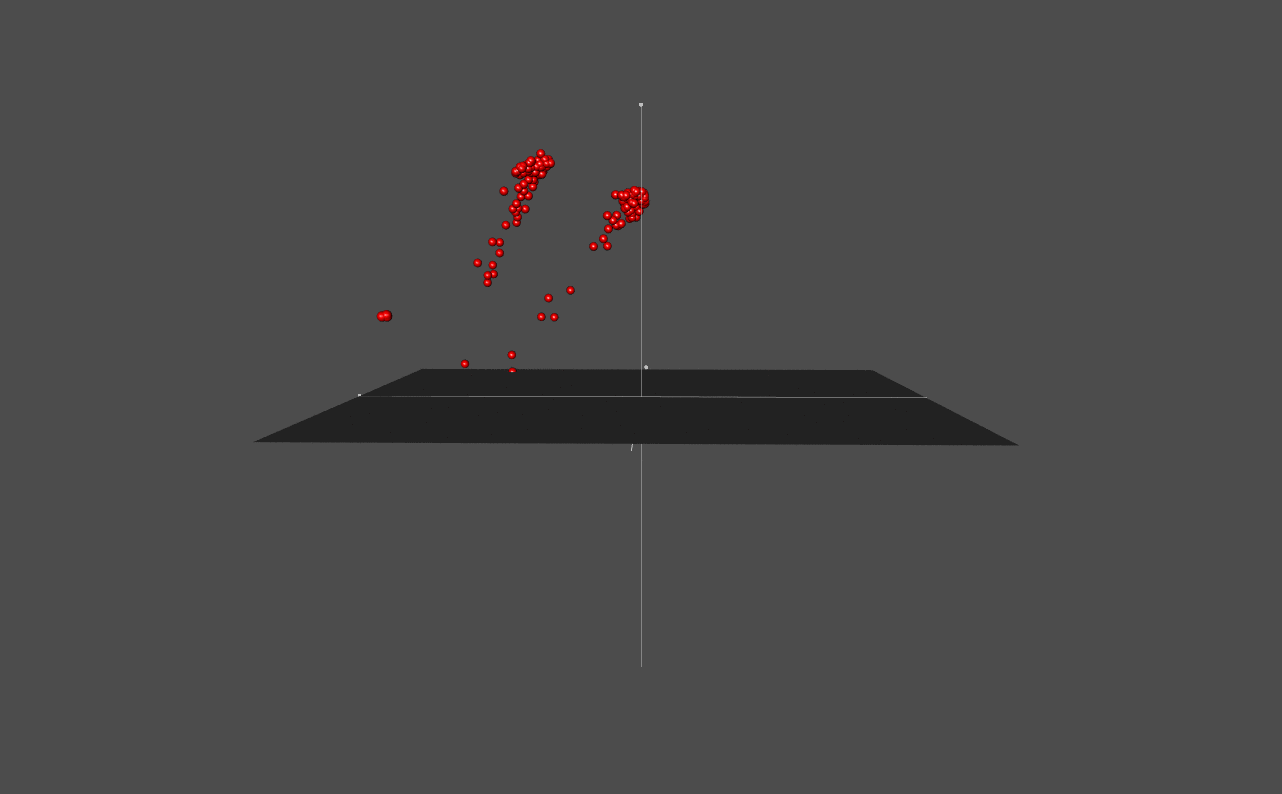

Supplement: Supplementary file 18 — Supplemental Video S2-(1) [file 41598_2018_37397_MOESM18_ESM.gif]

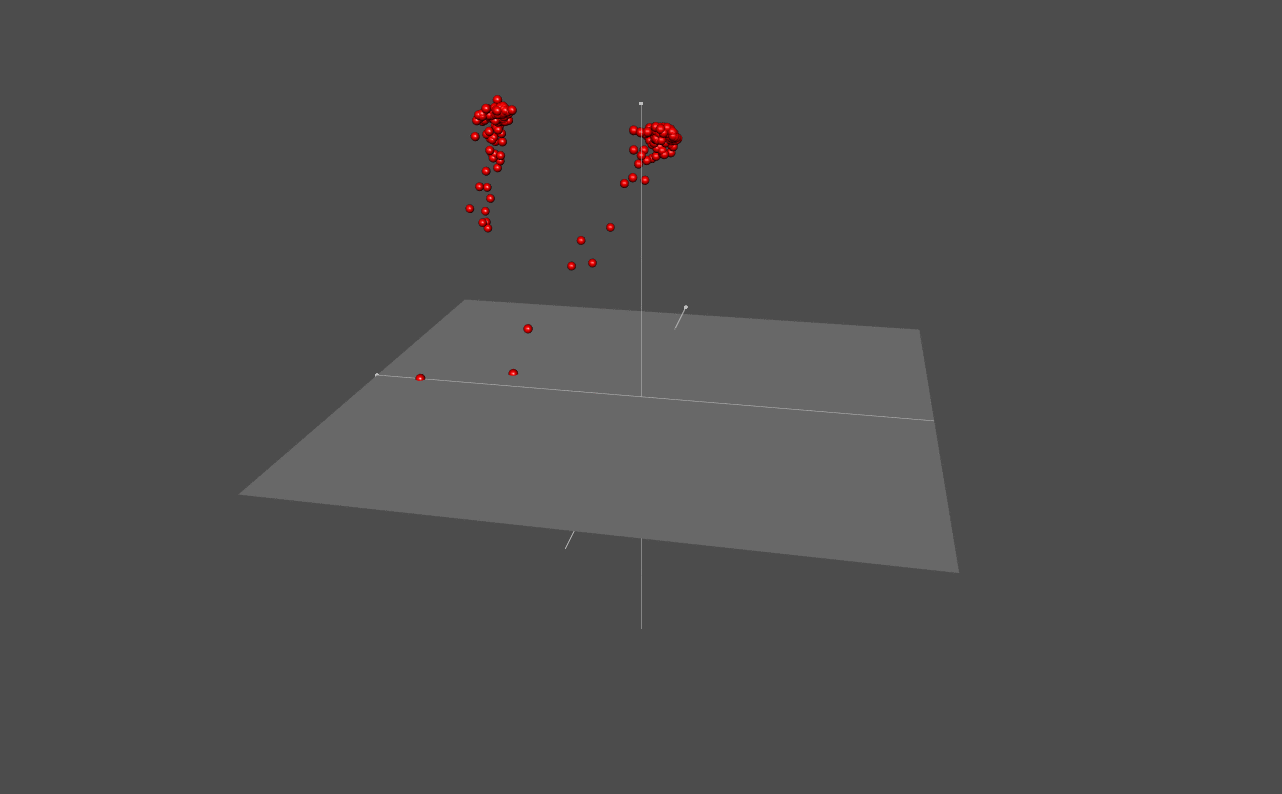

Supplement: Supplementary file 19 — Supplemental Video S2-(2) [file 41598_2018_37397_MOESM19_ESM.gif]
